# A Buffered Hexacyanoferrate Electrolyte for Thermogalvanic Heat Harvesting across Symmetric and Asymmetric Electrode Configurations

**DOI:** 10.1021/acsnano.6c04077

**Published:** 2026-07-09

**Authors:** Mohammad Zakertabrizi, Ehsan Hosseini, Mina Hosseini, Soheil Kavian, Ronald Sellers, Arian Zarriz, Matthew J. Powell-Palm

**Affiliations:** † J. Mike Walker ‘66 Department of Mechanical Engineering, 14736 Texas A&M University, College Station, Texas 77803, United States; ‡ Department of Materials Science and Engineering, 14736 Texas A&M University, College Station, Texas 77803, United States; § Department of Biomedical Engineering, 14736 Texas A&M University, College Station, Texas 77803, United States

**Keywords:** low-grade heat, thermogalvanic, energy harvesting, thermoelectric, Seebeck effect

## Abstract

Thermogalvanic waste-heat harvesting offers a promising route to the productive utilization of low-grade thermal energy, but current cells remain limited by low thermopower and power output. Here, we report a thermogalvanic electrolyte that inserts the canonical Fe­(CN)_6_^3–/4–^ redox cycle within an optimized NH_4_OH–CH_3_COOH alkaline environment. Our results show that the optimal mixture establishes differential formation energies between the hexacyanoferrate redox pair, thereby amplifying the configurational entropy difference between redox states and boosting the thermopower from 1.48 to 2.64 mV K^–1^ in a symmetric graphite cell, with a normalized power density of ∼1.5 mW m^–2^ K^–2^ sustained across Δ*T* = 10–50 K. We show that placing the optimized electrolyte within an asymmetric copper–graphite electrode setup leads to a hybrid mechanism of galvanic-thermogalvanic (gTg), which produces a total output substantially exceeding the sum of isothermal galvanic and electrode-analogous thermogalvanic cell outputs, while also demonstrating significantly extended operational longevity over isothermal discharge. A 50-cell proof-of-concept device mounted on a PC tower produces 42 V and 160 mW total output, of which 7 V and 37.6 mW are derived from the thermal contribution.

## Graphical abstract

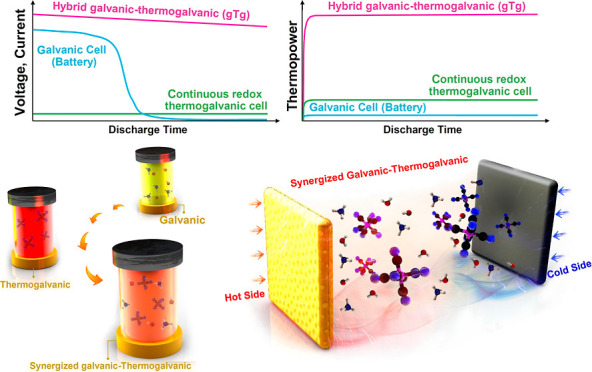

## Introduction

Management and recovery of low-grade (<100 °C) waste heat presents a landmark challenge to 21st-century industry, with an estimated 85 PWh yr^–1^ of energy rejected into the environment at these temperatures.^1^ The widespread adoption of industry-scale artificial intelligence, cloud computing, and other computationally intensive industrial processes promises only to increase this rate of waste,^2^ requiring ever-larger data centers with remarkable waste-heat signatures.^3–5^ This growing waste stream, coupled with those long associated with energy generation, manufacturing, transportation, and biological processes, creates a broad need for advancement in the productive utilization of low-grade heat.

Thermal-to-electrical energy conversion processes, in which electricity is generated in response to a temperature gradient Δ*T*, have long provided an aspirational route toward waste-heat recovery, but remain limited in thermopower (V/Δ*T*) and normalized power density (*P*_max_/Δ*T* ^2^) across process classes. Conventional solid-state thermoelectric processes are typically limited to thermopowers of μV K^–1^ and *P*_max_/Δ*T* ^2^ of order 0.1 mW m^–2^ K^–2^;^6^ continuous-output liquid thermogalvanic processes based on temperature-dependent redox reactions achieve thermopowers of order 1 mV K^–1^ and *P*_max_/Δ*T* ^2^ of order 1 mW m^–2^ K^–2^;^7^ and liquid thermodiffusive processes based on the movement of ions under a thermal gradient achieve thermopowers of order 10 mV K^–1^ and normalized power density of order 1 mW m^–2^ K^–2^, although with characteristically noncontinuous output.^8^

Given their considerably higher power metrics, reduced technological complexity, and potential for fabrication from sustainable resources,^9,10^ liquid cells incorporating thermodiffusion, thermogalvanic redox, or thermally regenerative cycles have attracted considerable recent attention, with remarkable increases in output reported over the past half-decade.^9,11–20^ Although thermally regenerative processes have demonstrated success in enhancing efficiency and power output, their requirement to function within a cyclic framework precludes continuous energy delivery. The limited output power of thermogalvanic cells and the operational complexity of thermally regenerative cells hinder their effectiveness as stabilizing energy sources for continuous operations such as Heating, Ventilation, and Air Conditioning (HVAC) systems in data centers.

Over the past decade, a variety of approaches to improving both output and efficiency in these systems has emerged. One overarching theme penetrating much recent work and serving to distinguish the opportunity space available to liquid-cell thermoelectric systems from their solid-state counterparts is the remarkable potential for hybridization of differing thermal-electrical processes. Foundational early work identified the potential of coupling thermodiffusive ionic processes in the bulk electrolyte with thermogalvanic redox processes at the electrolyte–electrode interface.^21^ Subsequent efforts have synergistically manipulated thermal and electrical transport,^9^ diffusion and redox kinetics at varying length scales, introduced principles of phase-change thermal energy storage,^22,23^ and leveraged complementary energy conversion phenomena such as photocatalysis^24^ to reach ever-improved performance. In parallel, systematic engineering of the electrolyte itself has emerged as a powerful route to enhancing thermopower without modifying electrode architecture. Cosolvents, chaotropic additives, and gel matrices that modulate solvation structure and ion transport.^7,25^

In this work, we demonstrate how operating the canonical Fe­(CN)_6_^3–/4–^ redox cycle within an optimized NH_4_OH/CH_3_COOH alkaline solution results in significant improvements to the thermopower. We use molecular-scale simulations to show that the higher charge density of the Fe­(CN)_6_^4–^ strongly attracts and retains NH_4_^+^ within its first and second solvation shell, while the coordination of NH_4_^+^ and water around the Fe­(CN)_6_^3–^ is disturbed by CH_3_COO^–^, together widening the configurational entropy difference between the two redox states leading to significantly higher thermogalvanic response in a symmetric graphite|graphite cell. Building upon this outcome, we subsequently integrated the optimized electrolyte into an asymmetric copper|graphite electrode configuration, combining galvanic and thermogalvanic mechanisms into one gTg cell. We show that the resulting hybrid system combines the high power density associated with galvanic discharge, originating from copper oxidation to the soluble [Cu­(NH_3_)_4_]^2+^ ammine complex and concurrent ferricyanide reduction at the graphite cathode, with continuous thermogalvanic energy conversion sustained under an applied thermal gradient. The gTg cell exhibits substantial thermal enhancement beyond the isothermal galvanic baseline and maintains prolonged output compared with the same cell operated under isothermal discharge conditions. Through systematic isothermal controls and compositional studies, we decouple the galvanic, thermogalvanic, and synergistic contributions to device performance. Furthermore, we demonstrate scalable device integration using a 50-cell proof-of-concept module, in which the thermal component harvests low-grade waste heat generated from a desktop computer. These findings establish solvation-environment engineering and hybrid electrochemical integration as complementary approaches for advancing efficient low-grade heat recovery technologies.

## Results

### Electrolyte Optimization

To optimize the electrolyte for thermogalvanic performance, we performed a single variable sweep over the electrolyte composition against the maximum potential difference normalized over temperature difference between graphite electrodes under open-circuit conditions (Figure 1A,B, with further details regarding electrolyte preparation and the experimental setup are provided in the Supporting Information). The use of NH_4_OH in previous studies showed potential for increasing the solubility and charge-storage capacity of the Fe­(CN)_6_^3–/4–^ redox pair, but has remained confined to redox flow batteries with little exploration of the underlying mechanism or broader applications.^25,26^ Our optimized electrolyte consists of three main components: a redox center (K_3_Fe­(CN)_6_), an alkaline medium (NH_4_OH), and a bond-breaking agent (CH_3_COOH). Figure 1A shows that at a constant CH_3_COOH concentration of 1 M, increasing the concentration of K_3_Fe­(CN)_6_ increases the thermopower across different concentrations of NH_4_OH, reaching a peak performance at 0.5 M K_3_Fe­(CN)_6_. Including CH_3_COOH, which Figure 1B shows to be integral to maximal thermopower, stabilizes thermopower at 1 M. While the Fe­(CN)_6_^3–/4–^ is shown to be best suited to perform in a near neutral pH range,^27^ we attribute this jump in thermopower (2.64 vs–1.4 mV K^–1 13^ to the balanced buffer state established with simultaneous presence of NH_4_^+^ and CH_3_COO^–^. We hypothesize that the optimal mix of NH_4_OH and CH_3_COOH maximizes the entropy difference between the two redox states as NH_4_^+^ replaces water and K^+^ in the close vicinity of the redox species (first solvation shell) while the CH_3_COO^–^ anion preferentially disrupts the NH_4_–H_2_O second solvation shell around Fe­(CN)_6_^3–^ over the Fe­(CN)_6_^4–^, due to the higher charge density of the latter imposing stronger Coulombic repulsion. This preferential disruption of the NH_4_^+^-populated solvation shell widens the configurational entropy difference between the two redox states. In parallel, CH_3_COO^–^ also disrupts bulk water structuring, enhancing the diffusion coefficients of both redox species and improving kinetic access to the electrode surface, as supported by our molecular dynamics results to come (Figure 2A–C).

**Figure 1:**
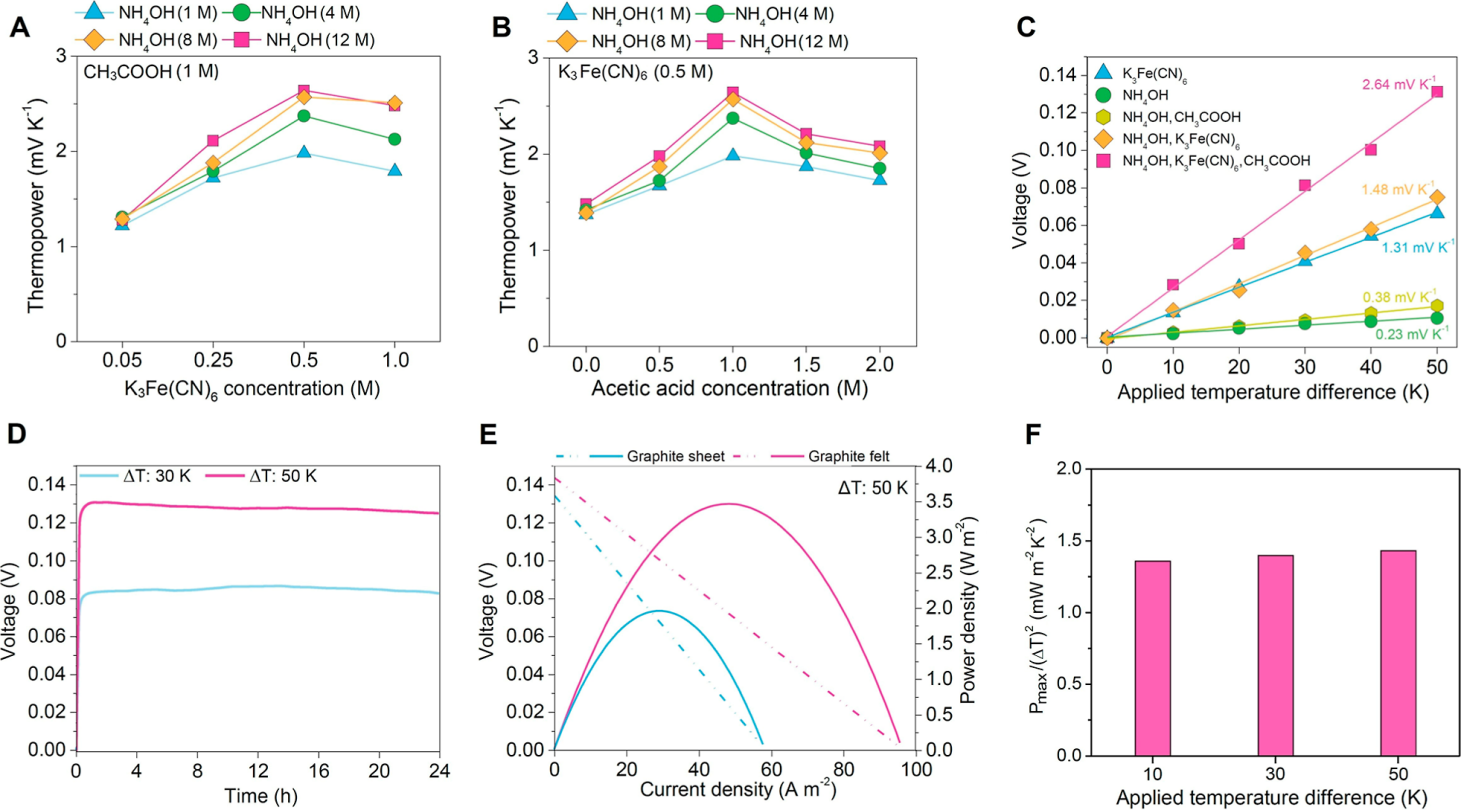
Optimization of a buffered Fe­(CN)_6_^3–/4–^ thermogalvanic electrolyte. (A) Thermopower as a function of K_3_Fe­(CN)_6_ and NH_4_OH concentration, with 1 M acetic acid and Δ*T* = 30 K (B) thermopower as a function of NH_4_OH and acetic acid concentration, with 0.5 M K_3_Fe­(CN)_6_ and Δ*T* = 30 K. (C) Thermogalvanic open-circuit voltage (*V*_oc_) and thermopower output were measured from each component of the optimized electrolyte in isolation. (D) *V*_oc_ under 30 and 50 K temperature gradients for the optimized electrolyte. (E) Voltage–current density–power density curves for cells with graphite sheet and graphite felt electrodes under Δ*T* = 50 K. (F) Normalized power density (*P*_max_/Δ*T* ^2^) measured under Δ*T* = 10, 30, and 50 K for optimized electrolyte. Origin of the Thermopower Enhancement.

**Figure 2:**
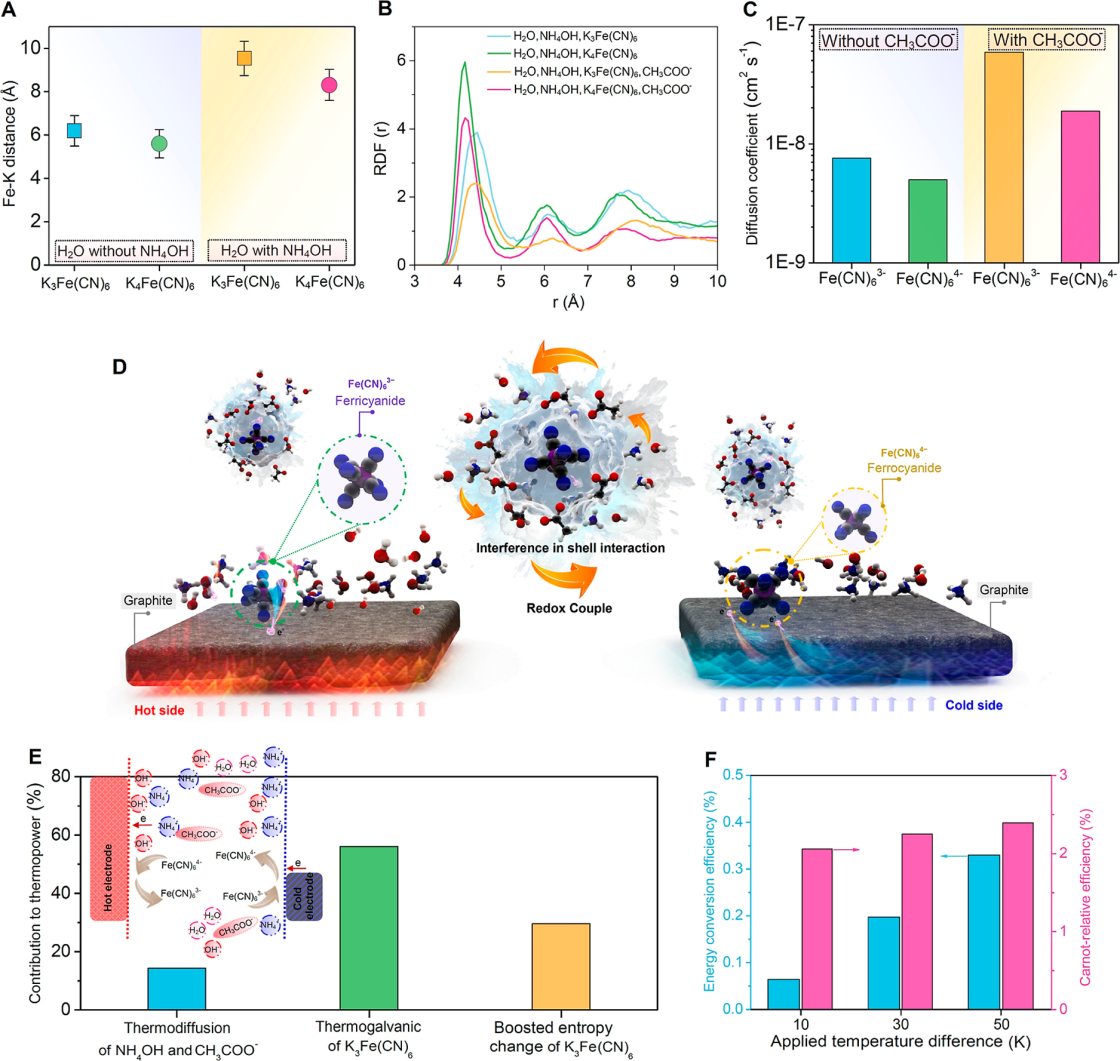
Dissecting the thermogalvanic contributions. (A) Evolution of the average Fe–K interaction distances within the [Fe­(CN)_6_]^3‑/4‑^ solvation shells upon the addition of NH_4_OH. (B) Radial distribution function of the electrolyte and (C) diffusion coefficients for the Fe­(CN)_6_^3–/4–^ redox pair obtained from molecular dynamics simulations. (D) Schematic of the thermogalvanic process within the graphite sheet|NH_4_OH–K_3_[Fe­(CN)_6_]–CH_3_COOH|graphite sheet cell. (E) Breakdown of chemical contributions to the total measured thermopower of the symmetric cell. (F) Energy conversion efficiency and Carnot-relative efficiency across Δ*T* values (10, 30, and 50 K).

We identified 12 M NH_4_OH, 1 M CH_3_COOH, and 0.5 M K_3_[Fe­(CN)_6_] as the optimal combination for thermogalvanic output. To eliminate the ambiguity often present in thermogalvanic preparation protocols, we emphasize that our system is intentionally initialized using only K_3_[Fe­(CN)_6_], rather than a conventionally premixed Fe­(CN)_6_^3–^/Fe­(CN)_6_^4–^ pair. By allowing the assembled cell to operate under open-circuit conditions for 120 h (further details in the Supporting Information), this procedure facilitates the in situ generation of a stable [Fe­(CN)_6_]^4–^ concentration, which completes the redox couple while minimizing the total K^+^ presence in the system. While our baseline testing confirms that conventionally premixed K_3_[Fe­(CN)_6_] and K_4_[Fe­(CN)_6_] solutions still deliver robust, highly stable thermopowers between 2.0 and 2.6 mV K^–1^ across various concentrations (Figure S1), allowing the system to reach a redox equilibrium from a pure K_3_[Fe­(CN)_6_] starting state consistently outperforms electrolyte with initial K_3_[Fe­(CN)_6_] and K_4_[Fe­(CN)_6_] presence. Furthermore, this 0.5 M concentration peak reflects both a thermodynamic and a solubility constraint. Consistent with existing literature identifying the 0.4–0.5 M range as optimal for entropy amplification in high-entropy solvents.^13,28^

Hereafter, all subsequent evaluations utilize this optimized formulation (0.5 M K_3_[Fe­(CN)_6_], 12 M NH_4_OH, and 1 M CH_3_COOH) strictly following its 120 h open-circuit equilibration. This hybrid electrolyte significantly outperforms its individual components, achieving nearly double the thermopower of a pure aqueous Fe­(CN)_6_^3–^ solution (Figure 1C). We hypothesize that this remarkable enhancement stems from a differential effect of the present ions on the solvation shell of the two-halves of the redox cycle. NH_4_^+^ preferentially populates the first solvation shell of Fe­(CN)_6_^4–^ via electrostatic pairing and highly directional hydrogen-bonding attractions between the nitrogen and hydrogen atoms, while CH_3_COO^–^ prefers actively disrupting the second solvation shell of Fe­(CN)_6_^3–^. Together, these synergistic effects widen the configurational entropy difference (Δ*S*_rxn_) between the oxidized and reduced states, directly amplifying the thermopower $S_{e}=\frac{\Delta S_{rxn}}{nF}$ . Furthermore, interactions within this alkaline environment uniquely drive the continuous, efficient cycling of the Fe­(CN)_6_^3–/4–^ redox pair (Figure S2). This active cycling is corroborated by FTIR spectroscopy. Notably, whereas a pure K_3_[Fe­(CN)_6_] solution exhibits sluggish Fe­(CN)_6_^4–^ formation over 120 h under a thermal gradient (Figure S3), the optimized electrolyte facilitates rapid, robust generation of the fully active redox couple (Figure S4).

Leveraging the identified optimal composition, we tested a prototype cell (electrolyte dimensions 1.2 × 1.2 × 3 cm; Figure S5) to monitor the open-circuit voltage at temperature gradients of 30 and 50 K, yielding sustained potentials of 88 and 134 mV, respectively (Figure 1D). These results demonstrate excellent thermopower stability over 24 h with small fluctuations, confirming the robustness of the stable redox system. Using this optimized electrolyte, we evaluated the impact of electrode architecture by comparing a 2D graphite sheet to a porous graphite felt (Figure 1E). While using the felt electrode maintains an open-circuit voltage similar to that of the planar sheet, its high-surface-area enables significantly higher current, effectively doubling the maximum power density (*P*_max_). Utilizing this superior porous felt architecture, we then tracked the system’s performance as the temperature gradient (Δ*T*) was scaled from 10 to 50 K (Figure 1F). The open-circuit voltage scaled linearly with the temperature gradient, while the maximum power density increased quadratically. As a result, the normalized power density (*P*_max_/Δ*T* ^2^) remained approximately constant.

### Proposed Mechanism

The optimized NH_4_OH–K_3_[Fe­(CN)_6_]–CH_3_COOH electrolyte exhibits significantly higher thermopower than its individual components or binary combinations (Figure 1C). We hypothesize that this enhancement originates from the restructuring of the solvation shells surrounding Fe­(CN)_6_^3–^ and Fe­(CN)_6_^4–^. Molecular dynamics simulations (MD) and density functional theory (DFT) calculations show that NH_4_OH and CH_3_COOH differentially modify the first and second solvation shells of the two redox states, leading to larger entropy differences and preferential ion partitioning, which together enhance thermogalvanic performance.

DFT calculations (Figure S9) reveal that the alkaline environment, with both NH_4_^+^ and CH_3_COO^–^ present, differentially modulates the formation energies of the [Fe­(CN)_6_]^3–^ and [Fe­(CN)_6_]^4–^. NH_4_^+^ replaces K^+^ around both redox species through directional N–H···N hydrogen bonding with the cyanide termini. This substitution is evidenced by an expansion of the Fe–K interaction distance (Figure 2A); the effect is slightly more pronounced for Fe­(CN)_6_^4–^ due to its greater negative charge, which facilitates stronger NH_4_^+^ recruitment into the first coordination shell (Figure S9). As NH_4_^+^ coordination around Fe­(CN)_6_^4–^ progressively increases, it becomes highly stabilized as (NH_4_)_4_[Fe­(CN)_6_]. Raman spectroscopy and FTIR analyses (Figures S3–S6) further confirm that NH_4_OH continuously promotes in situ reduction of Fe­(CN)_6_^3–^ to Fe­(CN)_6_^4–^ during operation.

Conversely, CH_3_COO^–^ selectively disrupts the second solvation shell of Fe­(CN)_6_^3–^. The higher-charge Fe­(CN)_6_^4–^ excludes CH_3_COO^–^ from its coordination environment through stronger electrostatic repulsion, whereas the lower-charge Fe­(CN)_6_^3–^ permits closer approach and disruption of its outer shell. Radial distribution function (RDF) analysis of the Fe (from Fe­(CN)_6_^3–/4–^)–N (from NH_4_^+^) interactions shown in Figure 2B, which represent the first and second solvation-shell layers, reveals this selective shell restructuring.

The Fe (from Fe­(CN)_6_^3–/4–^)–O (from H_2_O) RDFs shown in Figure S7B,C provide a complementary view of the solvation-shell restructuring illustrated in Figure 2B. Under pure aqueous conditions,H_2_O contributes significantly to the first and second solvation shells surrounding both Fe­(CN)_6_^3–^ and Fe­(CN)_6_^4–^. Upon addition of NH_4_OH and CH_3_COO^–^, the mixed H_2_O–NH_4_^+^ solvation shell is reorganized, resulting in reduced H_2_O ordering around the redox species. This behavior is consistent with the NH_4_^+^ RDF analysis (Figure 2B), which indicates preferential NH_4_^+^ incorporation into the solvation shell. At the same time, CH_3_COO^–^ selectively disrupts the second solvation shell of Fe­(CN)_6_^3–^, leading to increased disorder among the H_2_O molecules in its outer solvation shell. As a result, NH_4_^+^ promotes a more ordered local solvation structure around Fe­(CN)_6_^4–^, whereas CH_3_COO^–^ enhances disorder in the second solvation shell of Fe­(CN)_6_^3–^.

DFT configurations (Figures S8 and S9) show that NH_4_^+^ forms a strong solvation shell around both redox species, with a slightly stronger interaction with Fe­(CN)_6_^4–^ because of its greater negative charge. In contrast, CH_3_COO^–^ increases the difference in structural ordering between the solvation shells of Fe­(CN)_6_^3–^ and Fe­(CN)_6_^4–^. As a result, the formation-energy difference between the two redox states increases from 446 to 469 kJ mol^–1^ (Figure S9), leading to a larger entropy difference (Δ*S*rxn) and thereby enhancing the thermopower.

The restructuring of the solvation shells also improves ion transport. CH_3_COO^–^ increases the diffusion coefficients of both Fe­(CN)_6_^3–^ and Fe­(CN)_6_^4–^ (Figures 2C and S7), enhancing redox species accessibility at the electrode surface and reducing kinetic resistance. The ionic and effective electrical conductivities of the electrolyte are also significantly improved (Figures S10 and S11). Overall, this combination maximizes the thermodynamic driving force for thermogalvanic energy conversion while maintaining efficient charge transport throughout the electrolyte.

### Quantification of the Synergistic Enhancement

In addition to the widened entropy difference established above, every dissolved species (K^+^, NH_4_^+^, OH^–^, CH_3_COO^–^, and the [Fe­(CN)_6_]^3–/4–^ pair itself) also contributes a thermodiffusive term to the total measured thermopower. Disentangling these two channels is prerequisite to measuring the cooperative chemistry. We therefore compare the thermopower of the optimized electrolyte against the sum of the thermopower contributions of each constituent salt, measured individually under matched concentration, electrode, and Δ*T* conditions. Figure 2E reports this decomposition. Thermodiffusion of the background ions accounts for ∼14% of the measured thermopower; the bare thermogalvanic response of the redox couple, generated in situ from K_3_[Fe­(CN)_6_] via the spontaneous reduction identified in Figure S6, accounts for ∼57%; the remaining ∼29% is the synergistic excess. We attribute this excess to the differential solvation shell modification identified in the preceding section, where the alkaline NH_4_^+^/CH_3_COO^–^ environment simultaneously increases configurational disorder in the Fe­(CN)_6_^3–^ state and preserves order in the Fe­(CN)_6_^4–^ state, widening entropy difference beyond what either ion achieves on its own. Crucially, the stability of this is validated by 20 h continuous operational data (Figure S12). Under a constant load, the system maintains a highly stable voltage (∼80 mV) and current (∼6 mA). This persistent, long-term output contrasts sharply with transient ionic thermodiffusion, which quickly decays to near-zero under load. Such sustained performance confirms that the observed ∼29% power boost originates directly from a continuous increase in reaction entropy, functioning as a persistent thermogalvanic effect rather than a temporary ionic one. We calculated the thermal energy conversion efficiency η, defined as the ratio of output power to heat input (η = *P*_max_·d/(κ·A·Δ*T*)), where κ is the effective thermal conductivity of the cell (measured as κ_eff_ = 0.73 W m^–1^ K^–1^; Figure S5). The efficiency increases with Δ*T*, rising from 0.06% to 0.32% as Δ*T* expands from 10 to 50 K. The Carnot-relative efficiency (η_r_ = η/η_carnot_) reaches a peak of 2.5%, on par with other benchmark studies utilizing the Fe­(CN)_6_^4–^/^3–^ redox pair (Table S2).^7,29,30^

### The Galvanic-Thermogalvanic Cell

Conventional galvanic cells produce power via irreversible redox reactions that diminish as reactants are depleted and generally do not facilitate waste-heat conversion. Thermogalvanic cells, in contrast, produce power via reversible redox reactions driven by differing electrode temperatures, providing continuous conversion of thermal energy to electrical energy. However, conventional galvanic cells produce drastically more power than their thermogalvanic counterparts, presenting a trade-off between high power (galvanic) and operational longevity with thermoelectric conversion (thermogalvanic). To test whether the reversibility of thermogalvanic cycling can sustain the otherwise self-terminating galvanic process and overcome this trade-off, we coupled both mechanisms within a single cell by pairing our buffered Fe­(CN)_6_^3–/4–^ electrolyte with an asymmetric copper|graphite (Cu|G) electrode architecture (Figure 3A–C).

**Figure 3:**
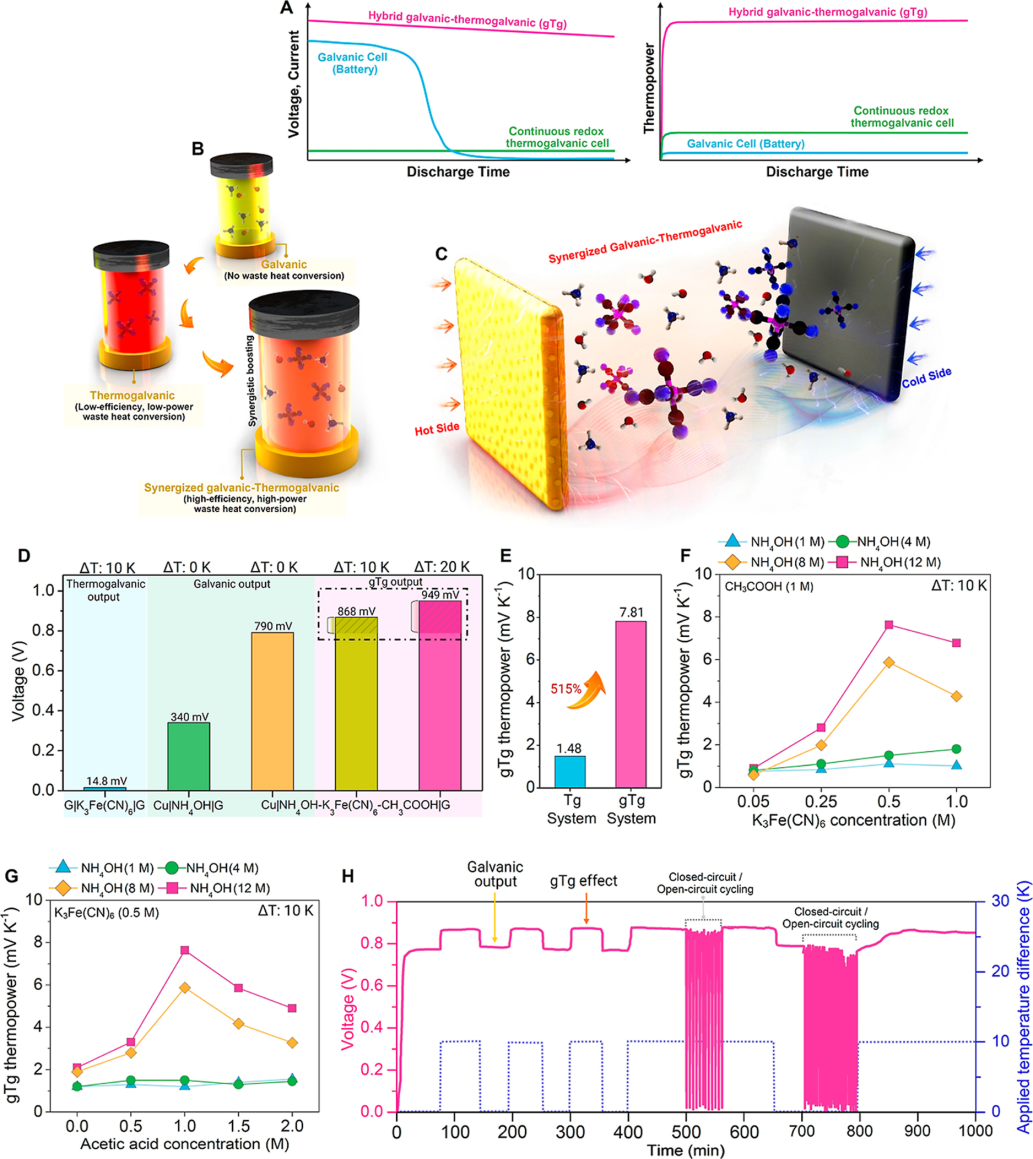
Galvanic-thermogalvanic performance. (A) Enhancement of the gTg architecture: integrating both mechanisms surpasses the isothermal galvanic and standalone thermogalvanic configurations in output potential, current density, and stability. (B) Comparison of gTg and conventional thermogalvanic output. (C) Simplified schematic of a gTg cell. (D) *V*_oc_ from different electrolyte mixtures under isothermal and nonisothermal (gTg) conditions; the dashed rectangle indicates the thermal contribution to total output. (E) Voltage response per unit temperature gradient for the symmetric G| NH_4_OH–K_3_[Fe­(CN)_6_]|G cell and the asymmetric Cu|NH_4_OH–K_3_[Fe­(CN)_6_]–CH_3_COOH|G cell under Δ*T* = 10 K. (F,G) Thermopower as a function of electrolyte concentration. (H) *V*_oc_ versus time under isothermal conditions and Δ*T* = 10 K, recorded over 17 h.

In this cell, the galvanic contribution arises from coupled electrode half-reactions: at the copper anode, Cu is oxidized and stabilized in solution as the soluble [Cu­(NH_3_)_4_]^2+^ ammine complex (Cu + 4 NH_3_ → [Cu­(NH_3_)_4_]^2+^ + 2e^–^), while at the graphite cathode, Fe­(CN)_6_^3–^ is reduced to Fe­(CN)_6_^4–^. The isothermal galvanic Voc against a graphite reference confirms the stability of the galvanic cell (Figure S13), and the corresponding open- and closed-circuit ion flows are illustrated schematically in Figure S14. Sustained galvanic output requires that Cu^2+^ released at the anode is captured as the soluble [Cu­(NH_3_)_4_]^2+^ ammine complex rather than precipitating as insoluble Cu­(OH)_2_/CuO, which would passivate the electrode, while maintaining the electrolyte within the stable operating pH window of the Fe­(CN)_6_^3–/4–^ redox couple (Figure S15).

Spectroscopic analysis (Figure S16) confirms that Cu–O stretching modes characteristic of CuOH/Cu­(OH)_2_ are suppressed during operation, indicating that ammine complexation outcompetes the passivation pathway. Comparison with alternative bases at matched concentrations (Figure S17) directly demonstrates the necessity of both functions. In contrast to (NH_4_)_2_CO_3_ and NH_4_OH, which supply NH_4_^+^ ions, NaOH and Na_2_CO_3_ exhibit negligible thermopower in the gTg setup, suggesting that the presence of NH_4_^+^ and activated CH_3_COO^–^ is critical for thermopower generation. These results underscore the advantages of the optimized electrolyte, which leverages high concentrations of NH_4_OH to provide the NH_3_ ligands necessary for ammine complexation, while employing a noncompeting acetate counteranion to stabilize the pH for [Fe­(CN)_6_]^3–^/[Fe­(CN)_6_]^4–^ redox activity. Within this composition, CH_3_COO^–^ plays an important role by maintaining the electrolyte at pH ∼10, within the stability window of the Fe­(CN)_6_^3–/4–^ redox couple, while also promoting ion transport through restructuring of the Fe­(CN)_6_ solvation shells (Figure S18).

We measured *V*_oc_ from the optimized Cu|electrolyte|G cell and subcombinations of the electrolyte components and electrode configurations under both isothermal and nonisothermal (Δ*T* = 10 K) conditions (Figure 3D). Under isothermal conditions, the full electrolyte produces a galvanic *V*_oc_ of 790 mV, arising from copper corrosion in the presence of ferricyanide. Upon applying a temperature gradient, *V*_oc_ increases to 949 mV; the difference (Figure 3D) represents the thermal contribution to total output. Critically, neither NH_4_OH nor K_3_[Fe­(CN)_6_] alone produces a comparable thermal enhancement, confirming that the full electrolyte synergy is required to activate the observed gTg mechanism.

To rule out the possibility that this enhancement arises from a temperature-dependent change in the galvanic potential rather than a synergistic contribution of the thermogalvanic mechanism, we measured *V*_oc_ under isothermal conditions across a range of uniform cell temperatures from 293 to 333 K (Figure S19A). The galvanic *V*_oc_ remains effectively constant (0.80 V) regardless of temperature, demonstrating that the galvanic potential is approximately temperature-independent over the investigated range. In contrast, when only one electrode is heated while the other is held at 293 K, *V*_oc_ increases monotonically with the applied gradient (Figure S19B). Subtracting the isothermal baseline from the gradient-driven voltage yields a net thermal voltage that scales linearly with Δ*T* at 7.81 mV K^–1^ (Figure S20), confirming that the enhancement is thermal gradient-driven.

This decoupling establishes a framework for interpreting the voltage enhancement quantitatively. Relative to the thermopower of the symmetric G|electrolyte|G cell (2.64 mV K^–1^; Figures 1D and S2), the asymmetric Cu|electrolyte|G architecture exhibits a substantially larger voltage response to applied temperature gradients (Figure 3E). The total gTg thermopower originates from three cooperative and thermally coupled processes: (i) the thermogalvanic entropy exchange of the Fe­(CN)_6_^3–/4–^ redox couple, enhanced by the differential NH_4_OH/CH_3_COO^–^ solvation environment and independently quantified as 2.64 mV K^–1^ in the symmetric G|electrolyte|G cell (Figure S12); (ii) the ionic thermodiffusion of the multispecies electrolyte, including NH_4_^+^, CH_3_COO^–^, K^+^, and OH^–^, which establishes temperature-dependent concentration gradients across the cell; and (iii) the thermally enhanced electrochemical reactivity of the Cu electrode (see Figure S21).

We propose that this asymmetric system operates via the following reaction: Cu + 2 Fe­(CN)_6_^3–^ + 4 NH_3_ → [Cu­(NH_3_)_4_]^2+^ + 2 Fe­(CN)_6_^4–^, while the continuous thermogalvanic output is sustained by the Fe­(CN)_6_^3–/4–^ redox pair. The thermal gradient also yielded a substantial boost in power output, with the inclusion of CH_3_COO^–^ enhancing the maximum power density under the applied gradient while having minimal effect under isothermal conditions (Figure S18). This modification not only introduces a supplementary galvanic component but also mitigates voltage attenuation during prolonged operation under a thermal gradient (Figure S22). Furthermore, the electrolyte composition optimized for the baseline thermogalvanic cell remains identically suited for the galvanic-thermogalvanic mechanism (Figure 3F,G).

We tracked the total voltage generated from the prototype cell over 16 h of continuous operation (Figure 3H). Upon assembly, Voc quickly reached a maximum of 790 mV under isothermal conditions at room temperature. Upon applying Δ*T* = 10 K between the electrodes (copper side: 308 K, graphite side: 298 K), Voc rose to 868 mV and remained stable. We then removed and reapplied the temperature gradient several times to confirm the consistency of this voltage change relative to the isothermal baseline, consistent with the solvation shell restructuring of the Fe­(CN)_6_^3–/4–^ redox couple described in the preceding section.

At the 500 min mark in Figure 3H, consecutive closed-circuit/open-circuit cycling was performed under a constant Δ*T* = 10 K. The cell was allowed to undergo voltage equilibration under open-circuit conditions and then subjected to power extraction (under a closed-circuit load) once the voltage plateaued; this procedure was repeated for 12 cycles. Based on FTIR results (Figures S4,S5 and S16), we attribute the observed behavior to temperature-dependent redox kinetics: higher temperature at the copper electrode accelerates copper oxidation and increases the concentration of Fe­(CN)_6_^4–^ at its surface under open-circuit conditions, increasing the potential relative to the graphite electrode. To confirm the thermogalvanic nature of these results, we performed identical load cycling tests under isothermal conditions (beginning at the 700 min mark in Figure 3H). In the absence of a temperature gradient, galvanic Voc decreased moderately each cycle, dropping 8% from its peak value over 100 min/35 cycles. Upon restoration of the temperature gradient, however, *V*_oc_ returned to its peak value and remained stable for the remainder of the observation period (200 min).

### Output and Operational Stability

To further disentangle the synergy at play, we systematically characterized the performance of the optimized cell (Cu|12 M NH_4_OH–0.5 M K_3_Fe­(CN)_6_–1 M CH_3_COOH|G) with and without an applied temperature gradient (Figure 4A). Under continuous discharge through a 333 Ω load for approximately 70 h, output under isothermal conditions at 293 K ceases after 30 h, consistent with galvanic depletion (Figure 4B). Under Δ*T* = 10 K (cold side 293 K; hot side 303 K), however, the cell discharges gradually and continuously, retaining more than half its initial output after 70 h and indicating effective conversion of thermal energy into electrical energy. This trend persists over five-day stability tests with alternating load cycles (Figure S23), where the gradient-driven cell maintains stable cycling while the isothermal cell degrades progressively; the working voltage further remains stable across the first 500 cycles for several electrolyte compositions (Figure S24). This degradation is attributed to standard copper anodic behavior, in which copper oxides, hydroxides, and Prussian blue analogs passivate the electrode surface and increase charge-transfer resistance. Importantly, isothermal discharge tests at elevated temperatures of 303 and 313 K (Figure 4B), both of which yield a higher average cell temperature than the Δ*T* = 10 K configuration, exhibit the same characteristic galvanic discharge to depletion, confirming the conclusions of the isothermal voltage measurements (Figure S17A): the enhanced output is attributable to the temperature gradient, not to elevated temperature per se.

**Figure 4:**
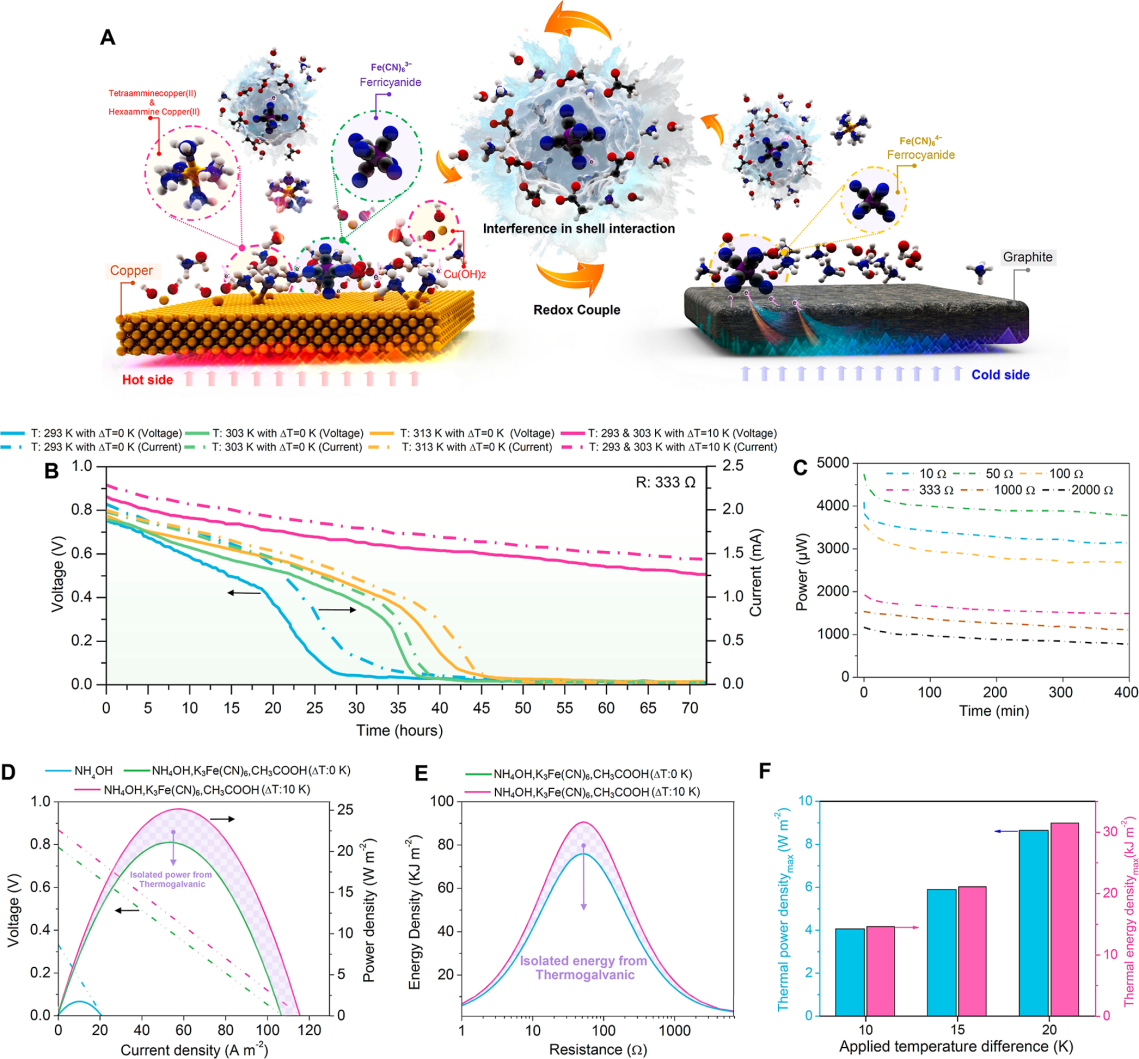
Output characterization of the optimized gTg cell. (A) Schematic of the thermally augmented galvanic contribution alongside the thermogalvanic redox cycle. (B) Cu|12 M NH_4_OH–0.5 M K_3_Fe­(CN)_6_–1 M CH_3_COOH|G cell discharging under isothermal conditions (293, 303, and 313 K) and under Δ*T* = 10 K with a 333 Ω load. (C) Output power under Δ*T* = 10 K for various external loads. (D) Voltage–current density–power density curves under isothermal and Δ*T* = 10 K conditions, compared to the isothermal output of the NH_4_OH base electrolyte. (E) Energy density under isothermal and gTg (Δ*T* = 10 K) operation for 1 h; the thermally augmented contribution is indicated between the two curves. (F) Thermal contribution to maximum power density and energy density at Δ*T* = 10, 15, and 20 K.

These results raise the question of whether the gradient effect could be purely convective, i.e., whether thermally driven internal flow simply refreshes the electrode surfaces. To test this, we introduced artificial flow (via a small propeller at 60 rpm) during isothermal discharge (Figure S25). Forced convection produced a rapid initial voltage drop to 0.2 V followed by a prolonged low-level plateau, a behavior qualitatively distinct from the gradient-driven cell, which maintains substantially higher voltage throughout the 70 h test. This indicates that while internal flow contributes to sustaining output (as previously reported by Gunawan et al.^31^), the thermal gradient provides an additional electrochemical driving force, namely the thermogalvanic voltage rooted in the entropy difference between redox states, as established in the electrolyte mechanism section, that forced convection alone cannot replicate.

We next measured the power output over a range of external loads from 10 to 2000 Ω (Figure 4C). Over a 400 min discharge cycle at Δ*T* = 10 K, the cell maintains steady output across load magnitudes, with exceptional steady power output (4000 μW) at 50 Ω. The current–voltage response (Figure 4D) shows a maximum output power density of 25 W m^–2^, approximately 19% larger than the same cell under isothermal conditions (21 W m^–2^). We thus estimate the overall thermal contribution as 4 W m^–2^. Peak energy density was observed at 51 Ω, at which the thermal contribution produced an additional 14.6 kJ m^–2^ relative to isothermal galvanic activity (Figure 4E).

While the symmetric graphite–graphite cell yielded a thermogalvanic power density of 0.1 W m^–2^ (consistent with the measured thermopower of 2.64 mV K^–1^), the asymmetric Cu|electrolyte|G cell demonstrated a total thermal enhancement of 4 W m^–2^ above its own isothermal galvanic baseline at Δ*T* = 10 K. This thermal enhancement encompasses both the thermogalvanic redox contribution, driven by the amplified entropy difference arising from the NH_4_^+^/acetate restructuring described in the preceding section, and thermally augmented galvanic kinetics; the two cannot be cleanly separated without further investigation. Figure 4F summarizes the combined thermal enhancement of power and energy densities: the thermal contribution nearly doubles from 4 to 8 W m^–2^, and the energy density increases from 15 to 30 kJ m^–2^ as Δ*T* is increased from 10 to 20 K.

### Multi-Cell gTg Device

To demonstrate the scalability of these high-power gTg cells, we combined 50 cells (each 1.2 × 1.2 × 3 cm) into a prototype waste-heat conversion device. The cells were arrayed in two configurations: all-series for maximum voltage, and five parallel rows of ten for balanced closed-circuit current–voltage. We placed the device over a cooling fan from a working PC, whose exhaust provides a stable 15 K temperature gradient with the surroundings. Output current was routed through a DC–DC buck converter for voltage stabilization and connected to a smartphone via USB-C (Figure 5A,B). The all-series configuration generated *V*_oc_ = 42 V, stable for 500 min; the same setup showed *V*_oc_ = 35 V with the fan off (Figure 5C). Under fan-on conditions, the system achieved a maximum peak total power output of 168.53 mW (gTg operation), sufficient to initiate charging of a connected Android smartphone. With the fan deactivated (galvanic output), peak power dropped to 130.9 mW (Figure 5D). By subtracting the galvanic contribution from the total output, the thermal contribution was determined to be approximately 37.6 mW and 7 V for the 50-cell series configuration.

**Figure 5:**
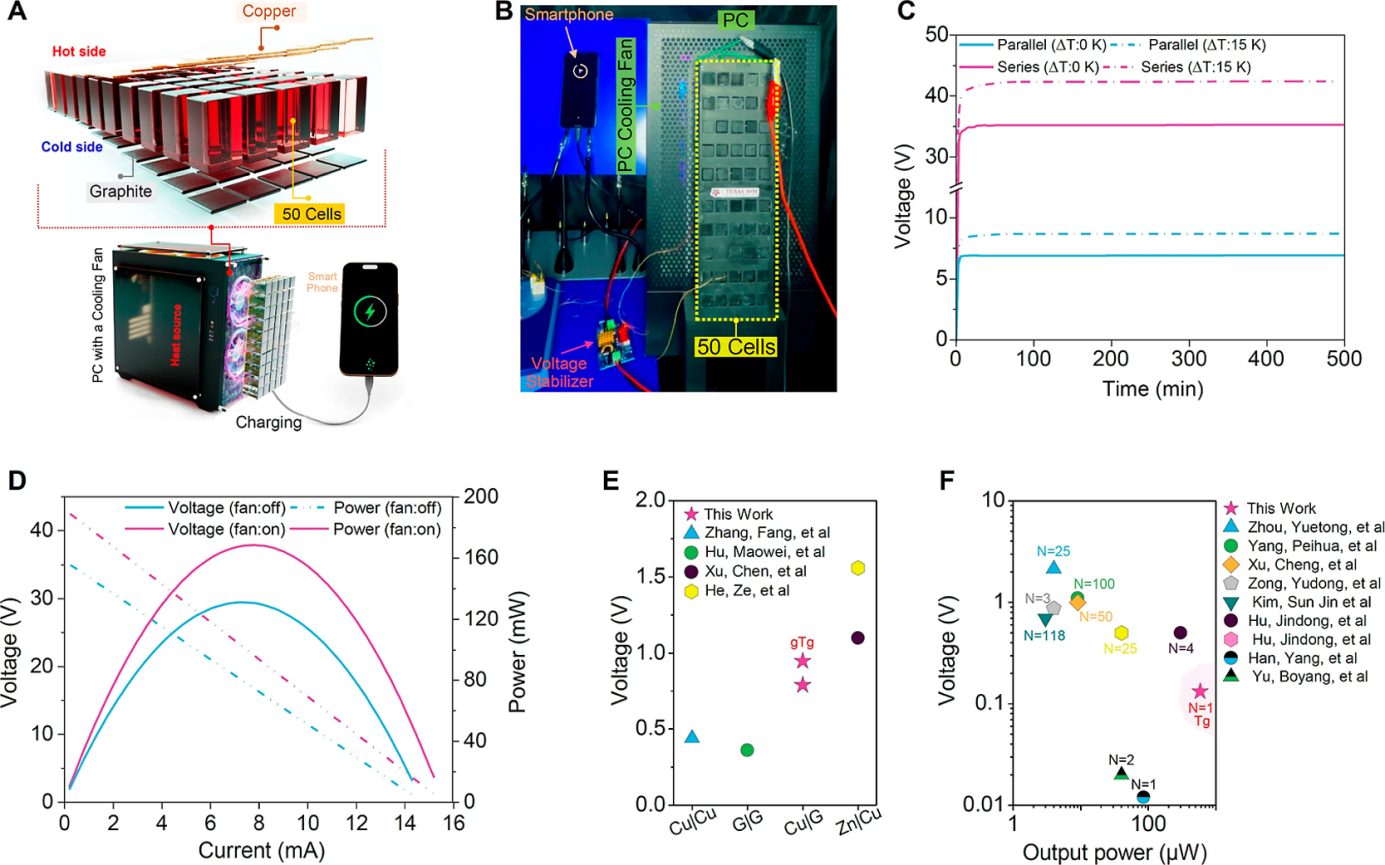
Proof-of-concept multicell gTg device. (A) Schematic of the 50-cells prototype and its mode of operation between the PC cooling fan (heat source) and ambient air (cold sink). (B) Assembled device: 5 parallel rows of 10 series-connected cells mounted over a PC cooling fan, routed through a voltage stabilizer to charge an Android smartphone. (C) *V*_oc_ with and without a temperature gradient for parallel and all-series configurations. (D) Voltage–current density–power density curves under 15 K temperature difference. (E,F) Comparison of the voltage–power performance of our system with previously reported energy-harvesting systems. (E) Comparison of the overall voltage output of our thermogalvanic–galvanic (gTg) system with reported galvanic and flow-cell systems. (F) Comparison of the purely thermogalvanic voltage and power output of our single graphite–graphite electrode cell with previously reported thermogalvanic systems.^18,32–41^

For a direct comparison, we contrasted the isothermal (galvanic) voltage of our prototype against previously reported galvanic and flow cells (Figure 5E). We then compared the purely thermogalvanic output generated by our single-cell graphite–graphite electrode configuration with the performance of other thermogalvanic systems reported in the literature (Figure 5F).^32–38^

## Conclusions

We have demonstrated that integrating the [Fe­(CN)_6_]^3–^/[Fe­(CN)_6_]^4–^ redox couple within a carefully optimized NH_4_OH–CH_3_COOH aqueous environment substantially enhances thermogalvanic performance. The buffer restructures the solvation entropy: the superior charge density of [Fe­(CN)_6_]^4–^ strongly sequesters NH_4_^+^ cations within its solvation shell, while simultaneously repulsing and redirecting CH_3_COO^–^ ions to disrupt the more labile solvation shell of the trivalent [Fe­(CN)_6_]^3–^ ions. This complementary action—maximizing order in the reduced state while inducing disorder in the oxidized state—widens the configurational entropy difference and enhances ionic diffusivity, yielding a thermopower of 2.64 mV K^–1^ and a normalized power density of ∼1.5 mW m^–2^ K^–2^ at Δ*T* = 50 K. The underlying principle, restructuring driven by the differing electrostatic affinities of secondary ions toward the two redox states, is consistent with the growing understanding that solvation shell asymmetry is a primary driver of thermopower in redox electrolytes,^7,20,42^ further underlining the solvation environment engineering as a general strategy for thermopower enhancement across redox couples.

Leveraging this electrolyte, we further demonstrated a galvanic-thermogalvanic architecture employing an asymmetric copper–graphite electrode configuration. The combined thermal contribution at Δ*T* = 10 K, which encompasses both the enhanced thermogalvanic redox cycle and thermally augmented galvanic kinetics, yields a total output enhancement of 4 W m^–2^ above the isothermal galvanic baseline, with the thermal gradient further extending operational longevity far beyond that of the same cell under isothermal conditions. The practical viability of this approach was validated by a 50-cell proof-of-concept device, which produced a collective power of 160 mW, with 37.6 mW produced as a result of the thermally augmented galvanic contribution combined with the thermogalvanic mechanism converting low-grade waste heat from a PC tower. These data suggest a blueprint from which to build thermal-to-electrical energy conversion capabilities into conventional electrochemical storage and discharge cells, opening a broad parameter space from which to approach the pressing waste-heat recovery challenges of the 21st century.

## Methods

### Materials

In this study, the following materials were used: Lab grade Ammonium Hydroxide, 50/50 wt. pure ammonia gas dissolved in purified water, and Acetic Acid Glacial 99% were sourced from Lab Alley. Potassium hexacyanoferrate­(III) (98%) was obtained from BTC. Graphite electrodes with an electrical resistivity of approximately 10 μΩ·m were purchased from Graphite Material Company Ltd. Additionally, pure copper electrodes were acquired from Canrd Co.

### Fabrication of the Prototype Cell

The prototype cell was 3D-printed using resin and assembled with graphite sheets (and an opposing 10 μm thick copper plate in the case of gTg cells) as electrodes, sealed with Sil-Poxy Rubber Silicone Adhesive. The electrode surface area in contact with the electrolyte was 1.44 cm^2^. Two commercial Peltier cells (4 × 4 cm) were used to generate and maintain the temperature at both electrodes. Two symmetric configurations were tested: graphite–graphite (G|G), in which identical graphite sheets were mounted on both faces, and copper–copper (Cu|Cu), in which 10 μm copper foil electrodes were mounted on both faces. The asymmetric galvanic-thermogalvanic (gTg) configuration employed one copper foil electrode on one face and one graphite sheet on the opposing face. Across all three configurations, the cell body, assembly procedure, electrode dimensions, and active surface area remained strictly identical; only the electrode material was varied. Temperature control was achieved through thermocouples placed directly between the electrodes and the Peltier cells. To prevent heat loss, the cell was sealed with thermal insulation tape. Copper tape and copper wire served as collectors, ensuring consistent results with low resistance and a reliable connection for measuring the cell’s output. Additionally, two standing fans were used as holders and to cool the Peltier cells (Figure S5). Generally, low temperatures were used without fans, and there was no operational pressure on the plates. In the absence of a temperature difference (i.e., when both side electrodes were at the same temperature), the average temperature was 296 K. When a temperature difference was applied, the cold side of the electrode was set to 293 K, with major tests conducted at hot-side temperatures of 303, 308, and 313 K.

We used a Keithley 2000 to measure voltage/current and recorded the data using a custom MATLAB code. To measure the open-circuit voltage, the Keithley 2000 was employed alongside the in-house MATLAB code. A resistance board was included in the circuit to measure working voltage and current during power extraction. In a closed-circuit/open-circuit cycle, the cell underwent voltage equilibration under a constant temperature difference until the open-circuit voltage plateaued. Depending on the procedure, the cell was either set to an ultralow resistance baseline by adjusting the resistance to zero or connected to a closed circuit with a predetermined resistance to simulate working conditions.

### Preparation of Electrolyte

To prepare the electrolyte, ammonium hydroxide (NH_4_OH) solutions were initially synthesized at 4 M concentrations 1, 4, 8, and 12 M with each solution having a total weight of 10 g. Using NH_4_OH as the primary solution, 10 g served as the basis for calculating the required concentrations of potassium ferricyanide and acetic acid. Potassium ferricyanide (K_3_[Fe­(CN)_6_]) was added to the NH_4_OH solutions in concentrations of 0.05 M (0.165 g), 0.25 M (0.823 g), 0.5 M (1.646 g) and 1 M (3.292 g) to investigate variations in electrochemical properties. Acetic acid (CH_3_COOH) with 99% purity was subsequently introduced in incremental amounts of 0.1 g (0.167 M), 0.5 g (0.833 M), 1 g (1.665 M), and 2 g (3.331 M). The mixtures were stirred continuously for 2 h until all components were fully dissolved.

Crucially, our system is initially prepared using only one-half of the thermogalvanic redox pair, K_3_[Fe­(CN)_6_]. Because of the NH_3_/NH_4_^+^ buffer system, instigated as a result of adding CH_3_COOH, the electrolyte sets toward a [Fe­(CN)_6_]^3–/4–^ equilibrium as the assembled cell is allowed to rest under open circuit conditions for 120 h. During this time, substantial Fe­(CN)_6_^4–^ presence is generated in situ to fully activate the redox cycle. All reported thermogalvanic measurements for this system, including long-term thermopower, voltage, power density and current were exclusively measured using electrolyte that has reached equilibrium under these conditions.

Figure S1 shows the operational behavior of the electrolyte using standard premixed concentrations of K_3_[Fe­(CN)_6_] and K_4_[Fe­(CN)_6_]. This figure provides crucial evidence of the system’s long-term functionality. Remarkably, whether operating with low or high concentrations of the [Fe­(CN)_6_]^3–/4–^ redox pair, the system consistently maintains a robust and highly stable thermopower ranging from 2.0 to 2.6 mV K^–1^. This sustained performance mapping proves that our optimized electrolyte design can reliably facilitate the necessary redox cycling over extended periods without severe performance degradation.

While these manually mixed solutions exhibited the same fundamental behavior, we specifically prefer the single precursor method due to the unique mechanics of our asymmetric system (gTg mechanism). In the specific case of the gTg cell, using an initial electrolyte mixture only containing the Fe­(CN)_6_^3–^ half of the [Fe­(CN)_6_]^3–/4–^ pair precursor is not just convenient; it is a deliberate design choice. Upon assembly, the asymmetric architecture immediately initiates a spontaneous galvanic reaction: the reactive copper anode undergoes oxidation (dissolving into the electrolyte as the [Cu­(NH_3_)_4_]^2+^ complex), which simultaneously forces the reduction of Fe­(CN)_6_^3–^ to Fe­(CN)_6_^4–^ at the inert graphite cathode. During the initial 120 h open circuit resting phase, this intrinsic galvanic discharging naturally and continuously generates the necessary Fe­(CN)_6_^4–^ species in situ. By the end of this equilibration period, both halves of the thermogalvanic redox couple are fully active. Consequently, under an applied thermal gradient, the asymmetric cell simultaneously delivers both galvanic and thermogalvanic effects by combining its intrinsic galvanic potential with a stable and continuous thermogalvanic output, without requiring manual premixing of K_4_[Fe­(CN)_6_] salt.

### Large-Scale Device

In this experiment, we harnessed the waste heat from a PC cooling fan to generate thermoelectric power. The prototype, made from resin, includes 50 cells with a total electrode area of 72 cm^2^. We tested two configurations: one with 5 parallel groups of 10 cells in series, and another with all 50 cells connected in series to achieve maximum voltage output. Each cell measures 1.2 cm by 1.2 cm and is 3 cm deep, with electrodes secured using silicone adhesive to prevent leakage.

The PC cooling fan transferred heat from the processor to the hot side of the thermoelectric cells, maintaining a stable temperature difference of approximately 15 K. This setup effectively utilized the waste heat from the fan to generate a measurable thermoelectric voltage. This demonstrates the potential of using readily available devices for sustainable energy applications.

We monitored the prototype’s performance under these conditions, gathering valuable data on its efficiency and potential applications in thermal management solutions for electronic devices. This approach underscores the feasibility of integrating thermoelectric technology with common electronic components to enhance energy efficiency and sustainability.

## Supplementary Material



## Data Availability

All data are available in the main text or the Supporting Information.

## Notes

Additional experimental and computational methods (molecular dynamics simulation protocols, density functional theory calculations, effective thermal conductivity measurements, electrochemical impedance spectroscopy, and FTIR, UV–vis, and Raman spectroscopy details); Supplementary Text elaborating on electrolyte equilibration, solvation-shell restructuring, the galvanic reaction mechanism, the operating pH window, and long-term cycling behavior; Figures S1–S25, covering control thermopower measurements, FTIR/Raman spectra, molecular dynamics simulation boxes and radial distribution functions, DFT formation-energy diagrams, electrical/thermal conductivity and impedance data, pH measurements, and extended discharge, cycling, and convective-flow stability tests; and Tables S1–S2, listing parameters/units and benchmarking thermopower, normalized power density, and Carnot-relative efficiency against literature values (PDF) (DOCX)
