# Supplementary material for: A Buffered Hexacyanoferrate Electrolyte for Thermogalvanic Heat Harvesting across Symmetric and Asymmetric Electrode Configurations
Source: ACS Nano. 2026 Jul 9;20(28):20176–87. doi: 10.1021/acsnano.6c04077 (PMC13394539; doi:10.1021/acsnano.6c04077)
Supplement: Supplementary file 1 [file nn6c04077_si_001.docx]

Supplementary Materials for

A Buffered Hexacyanoferrate Electrolyte for Thermogalvanic Heat Harvesting across Symmetric and Asymmetric Electrode Configurations

Mohammad Zakertabrizi${}^{1,\dagger}$, Ehsan Hosseini${}^{1,\dagger,*}$ , Mina Hosseini^1^, Soheil Kavian^1^, Ronald Sellers^1^, Arian Zarriz^1^, and Matthew J. Powell-Palm^1,2,3,*^

*^1^J. Mike Walker ’66 Department of Mechanical Engineering,* *Texas A&M University, College Station, TX 77803, USA*
*^2^Department of Materials Science and Engineering, Texas A&M University, College Station, TX 77803, USA*
*^3^Department of Biomedical Engineering, Texas A&M University, College Station, TX 77803, USA*
${}^{\dagger}$*Equal contribution*

*^*^Corresponding Authors:* [*powellpalm@tamu.edu*](mailto:powellpalm@tamu.edu)*,* [*ehsan.hosseini@tamu.edu*](mailto:ehsan.hosseini@tamu.edu)

**This Supplementary file includes:**

Methods and Experimental

Supplementary Text

Figs. S1 to S25

Table S1 and S2

# **Methods and Experimental**

## **Molecular Dynamics**

In this study, all simulations were performed using the Large-scale Atomic/Molecular Massively Parallel Simulator (LAMMPS) package and Materials Studio. We utilized molecular dynamics (MD) simulations to explore the synergistic effects in the electrolyte solution comprising various ionic species. The model was constructed in a three-dimensional periodic box, which included K^+^, Fe(CN)_6_^3-^, Fe(CN)_6_^4-^, NH_4_^+^, OH^-^, water molecules (H_2_O), acetate ions (CH_3_COO^-^), acetic acid (CH_3_COOH), and the [Cu(NH_3_)_4_]^+2^ complex. This combination ensures that the overall charge of the system remains neutral, which is a crucial condition for simulating real physical systems accurately. The dimensions of the periodic simulation box were carefully chosen to match the conditions of our experimental setup and were set to 79.14 Å × 79.14 Å × 39.57 Å (Fig. S7).

Our simulation is divided into two parts: one with the presence of acetate ions and acetic acid in the electrolyte, and the other without them. This allows us to investigate the effect of these species on the formation of the solvation shell around Fe(CN)_6_^3-^ and Fe(CN)_6_^4-^. We began by constructing an initial model of the electrolyte system using a forcefield that accurately represents the interactions between all components. Special attention was given to parameterizing Fe(CN)_6_^3-^ and Fe(CN)_6_^4-^, as these species involve complex coordination environments with transition metals and cyanide ligands. We referenced established parameters from Prampolini, Giacomo, et al. [1], which provided surface charge details and interaction potentials suitable for our MD simulations. The other species, including NH_4_^+^, OH^-^, CH_3_COOH, CH_3_COO^-^, and [Cu(NH_3_)_4_]^2+^, were parameterized using Universal Forcefield (UFF) [2, 3] for consistency across different species in the system, supplemented with data from the literature to ensure that their interaction potentials, bond lengths, and angles corresponded to experimentally validated values.

Before initiating the production simulation, it was critical to ensure that the system reached a stable equilibrium. This was achieved using the SMART method, a multi-step minimization algorithm that combines three key approaches: steepest descent, conjugate gradient, and Newton-Raphson methods. Steepest Descent was used to remove any large overlaps or instabilities in the system by gradually relaxing the structure while following the steepest slope of the energy surface. Following the initial relaxation, the conjugate gradient method was employed to refine the system further by optimizing the energy with respect to the forces acting on each atom, ensuring a more efficient minimization process than steepest descent. Finally, the Newton-Raphson method was used to achieve high precision in the final stages of the minimization process, ensuring that the system was thoroughly relaxed and ready for the dynamics phase.

The system was equilibrated in the NPT ensemble for 10 nanoseconds to allow both the pressure and temperature to stabilize. The target temperature was set to 298 K, and the pressure was maintained at 1 atmosphere using a Nose-Hoover thermostat and barostat [4]. This step ensured that the volume of the simulation box fluctuated to allow for natural density adjustments, leading to an accurate representation of the electrolyte solution. After achieving equilibrium in the NPT ensemble, we transitioned the system to the NVT ensemble for an additional 5 nanoseconds. In this ensemble, the volume was fixed, but the temperature was maintained at 298 K. The NVT ensemble provided a stable environment to analyze the dynamics of the system without the influence of volume fluctuations, making it ideal for subsequent analyses like diffusion calculations.

To gain insights into the structural organization of the ions and molecules in the electrolyte, radial distribution functions (RDFs) were calculated. RDFs provide information about the probability of finding a pair of atoms at a specific distance from each other, normalized by the average density of the system. In our analysis, we focused on the RDFs between key species such as Fe(CN)_6_^3-^/Fe(CN)_6_^4-^ and NH_4_^+^, [Cu(NH_3_)_4_]^+2^, and CH_3_COO^-^, as well as between other relevant ion pairs.

The transport properties of the ionic species were investigated by calculating the diffusion coefficients of the major components, including Fe(CN)_6_^3-^, Fe(CN)_6_^4-^, NH_4_^+^, and CH_3_COO^-^. The diffusion coefficients were determined by tracking the mean squared displacement (MSD) of each species as a function of time. The MSD is a measure of how far particles move from their initial positions, and it increases with time in a diffusive regime. The diffusion coefficient **𝐷** was calculated using the Einstein relation (1):

$$D=\frac{1}{6t}\left\langle\left( r\left( t \right)-r\left( 0 \right) \right)^{2} \right\rangle(1)$$

where t is the time, r(t) is the position of the particle at time t, and r(0) is its initial position. The slope of the MSD versus time plot in the long-time limit yields the diffusion coefficient for each species.

## **Density Functional Theory**

In this study, we employed Density Functional Theory (DFT) calculations [5] to investigate the interactions between ammonium (NH_4_^+^), acetate (CH_3_COO^-^), and the ferricyanide/ferrocyanide complexes Fe(CN)_6_^3-^ and Fe(CN)_6_^4-^. We put particular emphasis on understanding how coordination of NH_4_^+^ and CH_3_COO^-^ differentially modifies the solvation environments of the both redox states and thereby enlarges the configurational entropy difference between them.

Density Functional Theory (DFT) calculations [5] were performed to investigate the interactions between NH_4_^+^, CH_3_COO^-^, and the Fe(CN)_6_^3-^/Fe(CN)_6_^4-^ redox couple, with the goal of understanding how their coordination asymmetrically modifies the solvation shells of the two redox states. The calculations were designed to complement the molecular dynamics simulations using comparable ionic environments and electrolyte compositions.

The Fe(CN)_6_^3-^ and Fe(CN)_6_^4-^ complexes were treated as isolated species without K^+^ counterions, consistent with the dissociated state of potassium ferro/ferricyanide in aqueous solution. Multiple cluster configurations were constructed with systematically varied numbers of NH_4_^+^ ions (1–9) and CH_3_COO^-^ ions (1 or 3), corresponding to the “Without Water” configurations shown in Fig. S9. To maintain electroneutrality, OH^-^ ions were added according to the residual charge of each cluster. For example, the Fe(CN)_6_^3-^ system containing 9 NH_4_^+^ and 3 CH_3_COO^-^ ions carries a net charge of +3 and was balanced using 3 OH^-^ ions, whereas the analogous Fe(CN)_6_^4-^ system carries a net charge of +2 and was balanced using 2 OH^-^ ions. This approach reflects the chemical environment of the NH_4_OH/CH_3_COOH electrolyte, where OH^-^ acts as the natural counterion to NH_4_^+^. Additional configurations containing explicit water molecules together with NH_4_^+^, CH_3_COO^-^, and OH^-^ ions were also constructed (“With Water” panels in Fig. S9) to represent the fully hydrated buffered electrolyte and enable direct comparison with the pure aqueous systems.

Exchange–correlation interactions were described using the Perdew–Burke–Ernzerhof (PBE) generalized gradient approximation (GGA) functional. Grimme’s DFT-D3 dispersion correction with Becke–Johnson damping [D3(BJ)] was included to account for long-range van der Waals interactions. The Double Numerical plus Polarization (DNP) basis set was employed for all atoms with a real-space cutoff of 4.0 Å. All-electron calculations were performed without pseudopotentials to improve the description of the Fe center.

The exchange–correlation interactions were described using the Perdew–Burke–Ernzerhof (PBE) [6] generalized gradient approximation (GGA) functional. Grimme’s DFT-D3 dispersion correction with Becke–Johnson damping [D3(BJ)] was applied throughout to account for long-range van der Waals interactions [7, 8]. The Double Numerical plus Polarization (DNP) basis set was employed for all atoms with a real-space cutoff of 4.0 Å. All-electron calculations were performed without pseudopotential approximations to improve the accuracy of the Fe center description. For Fe(CN)_6_^3-^, spin-polarized calculations were conducted to account for the low-spin d^5^ electronic configuration of Fe(III) (S = 1/2) under the strong-field CN^-^ ligand environment. In contrast, Fe(CN)_6_^4-^ was treated using a spin-restricted approach corresponding to low-spin Fe(II) (d^6^, S = 0). Solvation effects were included implicitly using the Conductor-like Screening Model (COSMO) with a dielectric constant of ε = 78.4 to represent an aqueous environment, while also implicitly accounting for the electrostatic screening contribution of OH^-^ ions present in the NH_4_OH/CH_3_COOH buffer. Because OH^-^ follows the same differential solvation shell modification exclusion principle as CH_3_COO^-^, namely stronger exclusion from the highly charged Fe(CN)_6_^4-^ primary sphere by coulombic repulsion, it was not varied as an independent explicit parameter in the cluster calculations. Geometry optimizations were considered converged when the energy and force thresholds reached 1.0 × 10^-5^ Ha and 2.0 × 10^-3^ Ha Å^-1^, respectively. Self-consistent field (SCF) calculations employed a convergence tolerance of 1.0 × 10^-8^ Ha with a maximum of 200 iterations.

Formation energies were calculated using electronic energies without zero-point energy or thermal corrections according to:

$$E_{formation}=E_{complex}-\sum_{i} E_{isolated species} \left( 2 \right)$$

where E_complex is the total energy of the fully optimized Fe(CN)_6_ system containing NH_4_^+^ and/or acetate and/or water molecules, and E_isolated species represents the independently optimized energies of NH_4_^+^, acetate, water, and the isolated Fe(CN)_6_ species.

Effective Thermal conductivity

We used the hot-wire technique to measure the effective thermal conductivity of the electrolyte. While comprehensive descriptions of the method are available in other sources, a brief summary is provided here. A PFA-coated platinum (Pt) wire (length of 7.5 cm and diameter of 0.002794 cm) is fixed inside a 10 cm × 5 cm × 5 cm container with its two ends soldered to solid copper wires, with both ends later covered in insulating inert polymer to prevent current leak. The container is then filled with the electrolyte liquid and the wire functions as both a heater and a thermometer based on its resistance. On two sides, the soldered copper wires are connected to a Keithley 2425 as the current source and a Keithley 2000 as multimeter in a four-point configuration. Both devices are connected to and controlled by MATLAB through GPIB connections. The voltage is measured when a steady current (0.05 A) is applied for 0.9 seconds.

The transient temperature rise ΔT is calculated from the change in the resistance, relative to the initial resistance as shown in equation (3):

$$\Delta T=\frac{\Delta R}{R_{ref}\beta_{ref}} (3)$$

β_ref_ = 3.729 × 10^−3^ K^−1^ is the temperature coefficient of resistivity for the wire and R_ref_ is the resistance of the wire at 25 °C. The heat generation rate per unit length of the Pt wire (q) was calculated as shown in equation (4).

$$q=\frac{I^{2}R}{L} (4)$$

L is the length of the wire, R is the resistance of the wire at the initiation of the measurement and I is the fixed current from the source meter. The effective thermal conductivity is then calculated through equation (5) fit on the linear section of the diagram in the logarithmic time scale.

$$\kappa_{eff}=\frac{q/4\pi}{d\left( deltaT \right)/d\left( lnt \right)} (5)$$

The apparatus was verified by measuring the effective thermal conductivity of DI water and ethylene glycol (Fig. S10).

Evaluation of the gTg cell

Electrochemical impedance spectra (EIS) measurements were conducted in the frequency range 100 kHz to 0.1 Hz and a scan rate of 10 mV s-1. All electrochemical tests were performed on the electrochemical workstation (Bio-Logic, VMP3, SN 0897) with an Ag/AgCl as reference electrode. We calculate the ionic conductivity (S cm^-1^) using equation (6):

$$\sigma=\frac{L}{R_{b}A} (6)$$

Where L is the distance between the electrodes (cm), A is the cross-sectional area of the electrolyte/electrode interface (cm^2^) and R_b_ is the bulk resistance (Ω) extracted from the Nyquist plot (Fig. S11).

Ionic conductivity and the pH of the electrolyte was measured using SevenDirect SD30 conductivity meter (Fig. S15).

## **FTIR & UV**

We used Fourier transform infrared spectroscopy (FTIR) results from Bruker ALPHA-Platinum spectrometer and Spectrophotometer Genesys 10S UV-Vis to verify the proposed synergy mechanism within the aqueous system (Fig. S16). The infrared observations showed the presence of the redox pair in the working prototype electrolyte. We sampled as-prepared electrolyte, operating conditions and after 10 days to observe the changes to its chemistry (Fig. S3, S4 and S21). We observed a significant development at 2044 cm^-1^ that coincides with a drop in 2115 cm^-1^, associated with Fe(CN)_6_^4-^ and Fe(CN)_6_^3-^, respectively.[9, 10] This transformation coincides with the formation of [Cu(NH_3_)_4_]^2+^ evident from the rise in ~600 nm wavelength.

## **Raman spectroscopy**

Raman spectroscopy measurements were conducted to analyze the specific intermolecular interactions and the C≡N stretching modes of the cyanoferrate species within the various electrolyte environments. The spectra were acquired using a custom-built optical setup equipped with a continuous-wave 532 nm excitation laser. The optical pathway utilized a dichroic mirror to efficiently separate the excitation beam from the collected scattering signal, while an iris was employed for precise spatial filtering to optimize signal quality. The inelastically scattered light was collected using a transmission-mode configuration and subsequently directed into a high-resolution spectrometer (Ocean Optics HR) for spectral acquisition. All solution-phase measurements were performed at room temperature using standard 2 mL glass vials as transparent sample holders. To ensure a high signal-to-noise ratio for accurately resolving the cyanoferrate stretching peaks (in the 2000–2200 cm^-1^ region) and to precisely reflect the working conditions of the device, all tested solutions were prepared at the exact molar concentrations utilized in the operational electrolytes.

## **Theoretical methods**

Thermopower is defined as the ratio of the resulting electric field −𝑑𝑉/𝑑𝑥 to the applied temperature gradient 𝑑𝑇/𝑑𝑥 as in the equation (7).

$$S=\frac{-dV/dx}{dT/dx}=-\frac{dV}{dT} (7)$$

where 𝑆 is the thermopower, 𝑉 is the voltage, T is the temperature difference between the hot and the cold electrodes. However, this is only considering the voltage produced from the applied temperature difference. In our design, we deduct the voltage of the device in the ambient condition from the same device working under stable temperature difference to calculate the thermopower. *S* is also called thermopower and is used interchangeably with thermopower. In this work, we used the isolated voltage as the numerator. We note that for the asymmetric Cu|electrolyte|G architecture, this subtraction-derived quantity represents the total thermal voltage enhancement coefficient rather than an intrinsic thermopower, as it includes contributions from both thermogalvanic redox and thermally-augmented galvanic processes.

The energy conversion efficiency and Carnot relative efficiency

The energy conversion efficiency is defined as the ratio of input heat and maximum output power as in equation (8).

$$\eta=\frac{{Power}_{max}}{P_{heat}}=\frac{{Power}_{max}}{\left( \kappa_{eff}A\frac{\Delta T}{d} \right)} (8)$$

Where ${Power}_{max}$ is maximum output power, A is the single surface area of the electrode, d is the distance between two electrodes, $\kappa_{eff}$ is the effective thermal conductivity and ΔT is temperature difference between the two electrodes.

The maximum output power contribution of the combination of thermally-augmented galvanic process and the thermogalvanic (copper|graphite cell) contribution is calculated according to equation (9). We determined the net power density contribution by deducting the isothermal maximum output power (ΔT = 0 K) from the maximum output power density from the cell operating under a temperature gradient (ΔT = 10 K, ΔT = 15 K, ΔT = 20 K).

$${Power}_{max}=\frac{{{(V}_{oc}I_{sc})}_{\Delta T}-{{(V}_{oc}I_{sc})}_{isothermal}}{4} (9)$$

Where V_oc_ is the open-circuit voltage and I_sc_ is the short-circuit current.

The Carnot efficiency ($\eta_{c}=\frac{\Delta T}{T_{hot}}$) is the maximum possible efficiency for a heat engine. To evaluate how well a real device performs against this limit, the Carnot-relative efficiency ($\eta_{r}$) is used as a standard performance benchmark (12), calculated by equation (10):

$$\eta_{r}=\frac{\eta}{\eta_{c}}=\frac{{Power}_{max}/\left( \kappa_{eff}A\frac{\Delta T}{d} \right)}{\left( \frac{\Delta T}{T_{hot}} \right)} (10)$$

In Fig 1, the system is a symmetric graphite|graphite cell that operates entirely on the thermogalvanic effect. Because both electrodes are identical, there is no galvanic component present in the cell. Consequently, the isothermal baseline subtraction in Equation (9) is not meaningful here, as the maximum output power depends solely on the applied temperature gradient from the cell operating under a temperature gradient (ΔT = 10 K, ΔT = 20 K, ΔT = 50 K).

Table S1. Parameters, Symbols, and Units

| Symbol | Full Parameter Name | Unit |
| --- | --- | --- |
| S | Seebeck Coefficient / Thermopower (symmetric cell) | mV K^-1^ |
| S* | Total Thermal Voltage Enhancement Coefficient (gTg cell) | mV K^-1^ |
| ΔT | Applied Temperature Gradient | K |
| T | Temperature | K |
| V_oc_ | Open-Circuit Voltage | V |
| I | Current | mA |
| J | Current Density | A m^-2^ |
| P_max_ | Maximum Power Density | W m^-2^ |
| $\boldsymbol{Power}_{\boldsymbol{max}}$ | Maximum Output Power | mW |
| P | Power | mW |
| P_max_/ΔT^2^ | Normalized Power Density | mW m^-2^ K^-2^ |
| W | Energy Density | kJ m^-2^ |
| η | Energy Conversion Efficiency | % |
| η_r_ | Carnot-Relative Efficiency | % |
| κ_eff_ | Effective thermal Conductivity of Electrolyte | W m^-1^ K^-1^ |
| σ | Ionic Conductivity | S cm^-1^ |
| D | Diffusion Coefficient | cm^2^ s^-1^ |
| E_form_ | DFT Formation Energy | kJ mol^-1^ |
| d | Inter-Electrode Distance | cm |
| A | Electrode Active Surface Area | cm^2^ |
| R_ext_ | External Load Resistance | Ω |

## **Supplementary Text**

Fig. S1 shows the thermopower measured for electrolytes containing 0.45, 0.25, and 0.05 M K_3_Fe(CN)_6_ paired with 0.05, 0.25, and 0.45 M K_4_Fe(CN)_6_ (bottom and top axes, respectively) in 12 M NH_4_OH and 1 M acetic acid. The thermopower across all tested concentrations remains above ~2.0 mV K^-1^, demonstrating that the alkaline buffer environment fundamentally alters the organization and interactions of the redox pair.

The results reported in Fig. 1c are based on the experimental procedure detailed in Fig. S2. We report the thermopower of the cell after 120 hours of operation under open-circuit conditions (Voc). The time-dependent voltage and thermopower (voltage per temperature gradient, here ΔT = 30 K) were measured over 120 hours for a symmetric graphite-graphite cell utilizing a pure aqueous 0.5 M K_3_Fe(CN)_6_ electrolyte. The cell exhibited a slow voltage growth, eventually reaching ~40 mV (corresponding to 1.3 mV K^-1^). Our FTIR results confirm that, over this duration, the cell generates a sufficient amount of Fe(CN)_6_^4-^ to achieve a thermopower consistent with values reported in the literature. This slow voltage climb corresponds to the gradual formation of Fe(CN)_6_^4-^ following the initial charge accumulation from ionic diffusion, which produces the first ~15 mV (Fig. S2A).

This behavior is in stark contrast to the voltage climb observed in the symmetric graphite-graphite cell utilizing our optimized electrolyte (12 M NH_4_OH + 0.5 M K_3_Fe(CN)_6_ + 1 M CH_3_COOH). This optimized system reaches a significantly higher final open-circuit voltage of ~80 mV (corresponding to 2.64 mV K^-1^) at a much faster rate (Fig. S2B).

This accelerated kinetics is directly driven by graphite oxidation. Because the system utilizes carbon electrodes, the highly alkaline environment (high OH^-^ concentration) introduced by the concentrated ammonia significantly lowers the thermodynamic barrier for the oxidation of the electrode surface. Consequently, the graphite readily donates electrons to the Fe(CN)_6_^3-^ ions while forming oxygen-containing functional groups on the electrode surface and rapidly generating the necessary Fe(CN)_6_^4-^ species in the bulk liquid to establish the maximum thermopower. This specific chemical reduction of ferricyanide by graphite in alkaline environments has been recently verified in literature [11], perfectly corroborating our observation of accelerated Fe(CN)_6_^3-^ generation.

Fig. S3 depicts a comparison between the state of electrolyte within the G|K_3_Fe(CN)_6_|G cell at the beginning, after 48 hours and after 120 hours of operation. The CN-stretching band characteristic of Fe(CN)_6_^3-^ progressively diminishes while the Fe(CN)_6_^4-^ band emerges and grows, confirming time-dependent conversion of Fe(III) to Fe(II) within the cell. Fig. S4 shows the spectra collected after 48 and 120 hours of the G|optimized electrolyte|G cell operation. The CN-stretching feature near ~2115 cm^-1^ assigned to Fe(CN)_6_^3-^ at 48 h shifts to ~2040 cm^-1^, consistent with Fe(CN)_6_^4-^, by 120 h, indicating progressive reduction of the ferricyanide redox couple over extended operation.

Fig. S5 illustrates the single-cell test setup used for both symmetric (graphite-graphite and copper-copper) and asymmetric (copper-graphite) configurations. Across all tests, only the electrode materials were varied, while the dimensions and active surface area of the electrodes remained strictly identical.

Fig. S6 shows the Raman spectra used to investigate the structural evolution of the solvation environments surrounding the cyanoferrate ions. In the purely aqueous electrolyte, the spectra exhibit sharp, highly intense vibrational peaks, characteristic of a highly ordered, rigid hydrogen-bonded water network encompassing the redox-active species. Two key observations emerge from the buffered electrolyte spectra. First, the spectrum of K_3_Fe(CN)_6_ with NH_4_OH reveals a distinct peak at the Fe(CN)_6_^4-^ CN-stretch position (~2100 cm^-1^) that is entirely absent in the K_3_Fe(CN)_6_-only reference, directly confirming that NH_4_OH drives spontaneous in situ reduction of Fe(CN)_6_^3-^ and generates the Fe(CN)_6_^4-^ species required for the self-activating redox couple. Second, the fully optimized NH_4_OH/CH_3_COOH electrolyte after seven days exhibits pronounced C≡N stretching peak broadening and subtle baseline shifts. This severe spectral broadening provides direct physical evidence of structural disruption within the solvation shells. The widening of the vibrational bands physically reflects a vast distribution of diverse local bond microstates, consistent with the solvation restructuring described in the main text. NH_4_^+^, driven by stronger electrostatic affinity for the higher-charged Fe(CN)_6_^4-^ state, preferentially orders the primary coordination sphere of Fe(CN)_6_^4-^ through directed N–H···N hydrogen bonds with the cyanide nitrogen atoms [12]. On the other hand, CH_3_COO^-^ anions experience stronger repulsion from Fe(CN)_6_^4-^, preferentially disrupts the secondary solvation shell of Fe(CN)_6_^3-^. This complementary, charge-selective action widens the configurational entropy difference ΔS_rxn_ between the two redox states, directly serving as the thermodynamic driver for the remarkably enhanced thermopower and maximum output power observed in the optimized cell[13].

Fig. S7 shows the simulation box used in our molecular dynamics calculations to examine the microscale configurations and energy levels at the molecular level. The model contains NH_4_^+^, OH^-^, H_2_O, K_3_Fe(CN)_6_, K_4_Fe(CN)_6_ and CH_3_COO^-^ species. Figure S7B shows the radial distribution function (RDF) between the Fe center of Fe(CN)_6_^4-^ and the oxygen atoms of H_2_O, while Figure S7C shows the RDF between the Fe center of Fe(CN)_6_^3-^ and the oxygen atoms of H_2_O. In the NH_4_OH-containing electrolyte, both redox species exhibit well-defined first and second solvation-shell peaks, indicating a structured mixed H_2_O–NH_4_^+^ solvation environment surrounding the cyanoferrate ions. Upon the addition of CH_3_COO^-^, the RDF intensity decreases and the second solvation-shell features become less pronounced, reflecting reorganization of the local solvation environment. This effect is more pronounced for Fe(CN)_6_^3-^, where CH_3_COO^-^ increases disorder in the second solvation shell of Fe(CN)_6_^3-^, while the solvation shell surrounding Fe(CN)_6_^4-^ remains comparatively more ordered.

Expectedly, DFT simulations depict stronger interactions between NH_4_^+^ and Fe(CN)_6_^4-^ compared to NH_4_^+^ - Fe(CN)_6_^3-^. When acetate is added to the mix, it disrupts the NH_4_^+^ coordination around the redox ions differently: the effect is more pronounced for Fe(CN)_6_^3-^, where the lower charge density asserts a weaker repulsive force against the approaching CH_3_COO^-^ anion, allowing acetate to disrupt the secondary solvation shell. This is in contrast with the observations of the Fe(CN)_6_^4-^ solvation shell, where the repulsive force is strong enough to repel the CH_3_COO^-^ and retain near-uninterrupted NH_4_^+^ coordination shell. These observations confirm that the differential interactions of NH_4_^+^ and CH_3_COO^-^ with the redox ions enlarge the configurational entropy difference ΔS_rxn_ between the two states at the electrode interface (Fig. S8). We further tracked the formation energy as a function of NH_4_^+^/acetate composition (Fig. S9). For the Fe(CN)_6_^3-^/^4-^ redox pair, it shows the buffered alkaline environment of the optimized electrolyte stabilizing both species, with a stronger effect over Fe(CN)_6_^4-^ compared to Fe(CN)_6_^3-^, consistent with the differential solvation shell modification mechanism.

To quantify the differential modification effect, we evaluated the formation energetics of both redox states across aqueous and buffered environments (Fig. S9).

The Without Water panels (rows 1–3) isolate the direct formation energy between each ion and the surrounding NH_4_^+^/CH_3_COO^-^ ions, with the bulk aqueous environment represented implicitly by the COSMO dielectric. In the NH_4_^+^-only configurations (1^st^ row), formation energies become progressively more negative as NH_4_^+^ count increases from 1 to 9 for both ions. Fe(CN)_6_^4-^ consistently shows more negative values than Fe(CN)_6_^3-^ at every NH_4_^+^ count, reaching approximately −900 kJ mol^-1^ at 9 NH_4_^+^ compared to approximately −600 kJ mol^-1^ for Fe(CN)_6_^3-^. This persistent gap confirms that NH_4_^+^ binds more strongly to the higher-charge Fe(CN)_6_^4-^ center, reflecting its stronger electrostatic recruitment into the inner solvation shell. The progressively increasing formation energy of Fe(CN)_6_^4-^ with growing NH_4_^+^ coordination indicates that at sufficiently high NH_4_^+^ concentrations, the NH_4_^+^–Fe(CN)_6_^4-^ complex becomes energetically favorable for (NH_4_)_4_[Fe(CN)_6_].

The With Water panels (bottom row) show the baseline: formation energies of −1364 kJ mol^-1^ for Fe(CN)_6_^3-^ and −1810 kJ mol^-1^ for Fe(CN)_6_^4-^, yielding a formation energy gap of 446 kJ mol^-1^. The With NH_4_OH and CH_3_COO^-^, incorporating 9 NH_4_^+^ ions, 3 CH_3_COO^-^ ions, and explicit water molecules, show that the buffered environment further stabilizes both states but does so asymmetrically: Fe(CN)_6_^3-^ reaches −1658 kJ mol^-1^ (+294 kJ mol^-1^ relative to water alone), whereas Fe(CN)_6_^4-^ reaches −2127 kJ mol^-1^ (+317 kJ mol^-1^). The higher formation energy reflects the stronger NH_4_^+^ coordination with the Fe(CN)_6_^4-^ center, arising from the stronger local electric field and stronger N–H···N hydrogen bonding to the cyanide termini, while CH_3_COO^-^ and OH^-^, both anionic species, are preferentially excluded from its primary sphere by coulombic repulsion, thereby preserving the NH_4_^+^ coordination environment. In contrast, these anions can approach the Fe(CN)_6_^3-^ center more readily, disrupting its secondary solvation shell. Consequently, the formation energy gap widens from 446 kJ mol^-1^ in pure water to 469 kJ mol^-1^ in the buffered system. This enlarged energetic separation directly increases ΔS_rxn_ between the two redox states, consistent with the RDF data showing sharper coordination peaks around Fe(CN)_6_^4-^ and broader, more disordered peaks around Fe(CN)_6_^3-^, and thereby drives the observed enhancement in the thermopower.

We observed the effective electrical conductivity (σ_eff_)/resistance and effective thermal conductivity of the electrolyte at different temperatures, with respect to its composition (Fig. S10 and S11). The addition of acetic acid and K_3_[Fe(CN)_6_] significantly boosted the effective electrical conductivity of the electrolyte, as these components introduce a substantial number of ions to the mixture. We observed that the inclusion of CH_3_COOH significantly increases the ionic conductivity, highlighting its crucial role in enhancing the output. Further observations indicated that both effective electrical and thermal conductivity gradually increased with rising temperature[14].

As shown in Fig. S12, the optimized cell was subjected to a continuous discharge under a 10 ohm external load for 20 hours. Throughout this extended duration, the system delivered a remarkably stable and continuous output of both voltage and current. This sustained power delivery serves to show that the boosted entropy exchange is fundamentally driven by the continuous thermogalvanic redox cycling of the Fe(CN)_6_^3-^/^4-^ pair. If the generated potential were primarily governed by a thermodiffusion (Soret) mechanism, the ionic concentration gradients would rapidly deplete upon closing the circuit, causing the system to lose most of its output power within the first few minutes of closing the circuit. Therefore, the ability of our system to deliver continuous, non-decaying power over 20 hours unequivocally confirms that our electrolyte optimization successfully enhances the steady-state thermogalvanic conversion mechanism, rather than relying on short-lived thermal diffusion.

Fig. S13 illustrates the galvanic performance of our cells under isothermal conditions (ΔT = 0 K). It shows that achieving the maximum galvanic voltage depends on the concurrent presence of both NH_4_OH and K_3_[Fe(CN)_6_]. The data reveals a clear stabilization threshold: beyond a K_3_[Fe(CN)_6_] concentration of 0.25 M, the system reaches a steady, near-maximum galvanic voltage. Based on this electrochemical performance and established literature, the galvanic mode of the cell operates through two coupled half-reactions [11, 15]:

Anode:

Cu + 4NH_3_ → [Cu(NH_3_)_4_]^2+^ + 2e^-^

Cathode:

[Fe(CN)_6_]^3-^ + e^-^ → [Fe(CN)_6_]^4-^

Overall Reaction:

Cu + 2[Fe(CN)_6_]^3-^ + 4NH_3_ → [Cu(NH_3_)_4_]^2+^ + 2[Fe(CN)_6_]^4-^

During discharge, metallic copper is oxidized at the anode and stabilized in the solution as the soluble [Cu(NH_3_)_4_]^2+^ ammine complex, while [Fe(CN)_6_]^3-^ is simultaneously reduced at the cathode to [Fe(CN)_6_]^4-^. As the discharge progresses, the voltage naturally decreases as the [Cu(NH_3_)_4_]^2+^ complex accumulates and the [Fe(CN)_6_]^3-^ reactant is consumed.

In addition to providing the essential NH_3_ reactant for the anodic half-reaction, the ammonium species (NH_4_^+^) plays two critical, mechanistically distinct roles: (i) pH Buffering: The highly concentrated NH_4_OH is transformed into an alkaline buffer NH_4_^+^/NH_3_ with the addition of CH_3_COOH maintains the electrolyte at a stable pH of 9.87 (Fig. S15). This specific alkalinity falls perfectly within the operational stability window of the [Fe(CN)_6_]^3-^/[Fe(CN)_6_]^4-^ redox couple, while remaining safely above the acidic threshold where Cu(OH)_2_ passivation of the copper electrode would typically dominate. (ii) Ligand Supply for Cu^2+^ Complexation: The free NH_3_ in equilibrium with NH_4_^+^ (NH_4_^+^ ⇌ NH_3_ + H^+^) directly complexes with newly formed Cu^2+^ to yield the highly soluble [Cu(NH_3_)_4_]^2+^ ammine. This preferential complexation prevents the precipitation of insoluble Cu(OH)_2_ or CuO layers that would otherwise passivate the anode and halt cell operation.

Fig. S14 presents a comprehensive schematic mechanism of the gTg cell. Under open-circuit conditions (Fig. S14A), the system establishes its initial resting potential, governed by the spatial distribution of the multi-species electrolyte and the applied thermal gradient. Upon closing the circuit under an external load (Fig. S14B), the cell transitions into a dynamic steady state characterized by continuous electron flow.

The pH measurements (Fig. S15) show that while the main component, NH_4_OH, is very basic, the subsequent addition of K_3_Fe(CN)_6_ and acetic acid reduces the pH. The pH of the final composition remains basic at 9.87. Measurements after 30 hours of operation showed a consistent decline in the pH for all examined electrolytes, with the optimized electrolyte reaching close to 8. This is a result of the OH^-^ ions gradually exiting the electrolyte through reaction with copper at the final phase of its oxidation process.

The operating pH of the gTg electrolyte must simultaneously satisfy two competing chemistries: (i) stability of the Fe(CN)_6_^3-^/^4-^ redox couple, which is highest near neutral pH and degrades through CN^-^ release at strongly acidic or alkaline conditions,[16] and (ii) preferred production of the soluble [Cu(NH_3_)_4_]^2+^ ammine complex, which is the dominant Cu(II) species only in the alkaline range pH ≈ 9-11. Below pH ≈ 8, Cu(OH)_2_(s) precipitates and passivates the electrode; above pH ≈ 11, the soluble cuprate [Cu(OH)_4_]^2-^ and CuO(s) become competitive [17, 18]. The intersection of these two constraints defines a narrow optimal window centered at pH ≈ 9.5–10. Our optimized electrolyte sits at pH = 9.87, within this window, and Fig. S15 confirms experimentally that the maximum thermopower and short-circuit current are obtained at this pH.

Fig. S16 compares the FTIR spectra of the electrolyte across varying environmental and operational conditions after 10 days of aging and cell operation. Most critically, the spectra acquired from the active cell under continuous load and thermal cycling distinctly exhibit the preserved characteristic C≡N stretching frequencies corresponding to both the [Fe(CN)_6_]^3-^ and [Fe(CN)_6_]^4-^ states. This serves to show the sustained reversibility and long-term chemical stability of the electrolyte.

Fig. S17 demonstrates the voltage generated from the [Fe(CN)_6_]^3-/^ ^4-^ redox system under an applied thermal gradient using different alkaline electrolytes. Among all investigated alkaline media, the ammonia-based electrolyte (NH_4_OH) exhibits the highest thermopower performance under thermal-gradient conditions. This enhanced response is primarily attributed to the solvation restructuring that NH_4_^+^ uniquely enables: unlike monovalent alkali cations (Na^+^, K^+^) or carbonate-based counterions, NH_4_^+^ forms directed N–H···N hydrogen bonds with the cyanide termini of the cyanoferrate species and does so with markedly stronger affinity for the higher-charged Fe(CN)_6_^4-^ (4^-^) state than for Fe(CN)_6_^3-^ (3^-^). This charge-driven preferential ordering of the reduced state's coordination sphere widens the configurational entropy difference ΔS_rxn_ between the two redox states, directly amplifying the thermopower. Alternative bases, including NaOH, Na_2_CO_3_, lack either the hydrogen-bonding geometry or the non-competing counter-anion required to sustain this asymmetric coordination, resulting in lower thermopower.

In addition, the ammonia-based electrolyte exhibits superior compatibility with acetic acid (CH_3_COOH), whose acetate anion (CH_3_COO^-^), experiencing stronger repulsion from the [Fe(CN)_6_]^4-^, is pushed towards the [Fe(CN)_6_]^3-^ to disrupt its secondary solvation shell without causing significant disruption to NH_4_^+^-ordered primary sphere of Fe(CN)_6_^4-^. This charge-selective secondary-shell disruption further widens ΔS_rxn_ beyond what NH_4_^+^ substitution alone achieves and additionally enhances ion diffusion and thermally driven electrochemical activity, ultimately resulting in the significantly enhanced thermopower.

This behavior is further corroborated in Fig. S18, where the optimized electrolyte composition containing NH_4_OH, K_3_[Fe(CN)_6_], and CH_3_COOH exhibits a noticeable increase in maximum power density under non-isothermal conditions (ΔT = 10 K) compared with isothermal operation (ΔT = 0 K). The enhancement in power density directly confirms the contribution of the gTg setup, where the applied thermal gradient augments the total electrochemical output of the cell. The observed increase in power generation demonstrates that thermal energy actively contributes to charge transport and redox kinetics, thereby boosting the overall electrical performance of the optimized electrolyte system.

In order to rule out the effect of raised temperature on the voltage output, we evaluated the cell under isothermal conditions with the electrodes kept under fixed temperatures of 293, 303, 313 and 323 K. The results are shown in Fig. S19, depicting very little change in voltage as the temperature in the cell is raised. In the gTg setup, the graphite electrode was kept at 293 K, the temperature of the copper electrode is raised from 293 K (isothermal) to 323 K (ΔT = 40 K). When deducted from the corresponding voltage of the isothermal setup, the net voltage shows a constant increase that resembles linear growth, with the gradient of 7.81 mV K^-1^ (representing the combined thermal voltage enhancement, not an intrinsic thermopower) (Fig. S20).

Time-dependent voltage and thermopower (defined as the generated voltage per unit temperature gradient, here ΔT = 30 K) were systematically measured. Replacing the inert symmetric graphite–graphite (G|G) electrodes with symmetric copper–copper (Cu|Cu) electrodes fundamentally alters the voltage evolution over the same timeframe (Figs. S2 and S21).

As shown in Fig. S21, utilizing a 0.5 M K_3_[Fe(CN)_6_] solution with symmetric Cu|Cu electrodes in both pure aqueous and optimized electrolytes initially produces a small, transient galvanic voltage under isothermal conditions. This baseline potential likely originates from spontaneous interfacial reactions between the electrolyte and the reactive copper surface, eventually reaching a near-stable plateau before gradually decaying after 120 minutes.

However, the isolated thermopower of this symmetric Cu|Cu configuration is relatively modest at approximately 1.2 mV K^-1^, noticeably lower than that of the reference G|G cell. While the absolute thermopower derived from this symmetric Cu|Cu system is not directly comparable to the large voltage outputs observed in the asymmetric configuration (which is primarily driven by a dominant galvanic potential).

To evaluate the practical energy storage and continuous delivery capabilities of our system, we recorded the voltage and current output during extended charging and discharging phases under a 333 Ω load (Fig. S22). As expected, the baseline pure NH_4_OH electrolyte exhibits a rapid depletion of both voltage and current, confirming its inability to sustain continuous power generation on its own.

In stark contrast, the optimized multi-component electrolyte (NH_4_OH, K_3_[Fe(CN)_6_], and CH_3_COOH) delivers a highly stable, prolonged discharge profile even under isothermal conditions (ΔT = 0 K). Crucially, when a thermal gradient (ΔT = 10 K) is applied to this optimized system, both the voltage and current plateaus are noticeably elevated throughout the entire discharge phase. This sustained enhancement clearly illustrates the synergistic gTg effect, where the thermal gradient continuously augments the fundamental galvanic output power.

The profound impact of thermally driven mass transport on the long-term durability of the cell is ultimately demonstrated in a five-day cyclic load test (Fig. S23). The cell was subjected to alternating “Load ON” and “Load OFF” periods. Under both isothermal (ΔT = 0 K) and non-isothermal (ΔT = 10 K) conditions, the cell exhibits excellent electrochemical reversibility, consistently recovering its open-circuit potential during the resting phases. Naturally, the non-isothermal system maintains a superior, stable operating voltage across five days of operation.

A highly significant mechanistic insight is revealed by the behavior of the isothermal cell when operating without intermittent physical agitation (represented by the dashed line in Fig. S23). Without gentle mechanical shaking before each cycle, the isothermal system suffers a catastrophic voltage collapse by Day 3. This rapid degradation is a classic signature of severe concentration polarization, caused by the localized buildup of bulky [Cu(NH_3_)_4_]^2+^ products and depletion of active cyanoferrate species at the electrode interfaces. Conversely, the system operating under a 10 K thermal gradient sustains continuous, non-decaying operation for all five days without requiring any external mechanical agitation.

To further validate the exceptional reversibility and cycle life of the prototype cell (Copper | electrolyte | graphite), we monitored the working voltage over 500 continuous load cycles (Fig. S24).When utilizing the baseline NH4OH electrolyte, the cell exhibits a relatively low starting voltage that steadily degrades over the 500 cycles, regardless of whether it operates under isothermal (ΔT = 0 K) or non-isothermal (ΔT = 10 K) conditions. In distinct contrast, the optimized multi-component electrolyte (NH_4_OH, K_3_[Fe(CN)_6_], and CH_3_COOH) dramatically elevates the operating voltage and demonstrates vastly superior capacity retention. Most importantly, the optimal configuration, combining the synergistic electrolyte formulation with an applied 10 K thermal gradient, delivers the highest and most remarkably stable working voltage across all 500 cycles. The continuous thermal gradient actively suppresses performance decay over hundreds of cycles, confirming that the sustained gTg mechanism and thermally induced mass transport effectively maintain robust electrochemical kinetics over an extended operational lifespan.

We also explored the role of internal flows—like convection—on the voltage using constant turbulence from a small propeller (revolving at 60 rpm) placed in the middle of the cell (see Fig. S25). We hypothesize that these flows, normally a result of the temperature difference, can disturb the increasing presence of oxidation products near the copper electrode, thus acting as a recovering factor. We found that the output follows the general path of the galvanic setup, with a quick drop in the voltage before the 40-hour point. However, this drop occurs much earlier, around the 10-hour mark, yet the device maintains a higher voltage for an extended period. We hypothesize that the mixing flow provides a higher chance for the present ions to make contact with copper as the constant flow clears the oxidation products from around the copper electrode. Nevertheless, the disturbance from the flow also hinders the oxidation process, leading to a stable but very limited voltage. This indicates that the impact of applying a temperature gradient, gTg, extends beyond generating flow to remove oxidation products that could obstruct the oxidizer’s access to the metal electrode.

## **Supplementary Figures**

**Fig. S1.**

Thermopower as a function of varying K_3_Fe(CN)_6_/K_4_Fe(CN)_6_ composition ratio in the optimized NH_4_OH-CH_3_COOH electrolyte.

**Fig. S2.**

Symmetric-cell control measurements isolating the contributions to the asymmetric gTg total thermal response. Voltage and Thermopower time series of (A) G|K_3_Fe(CN)_6_|G, (B) G|electrolyte|G.


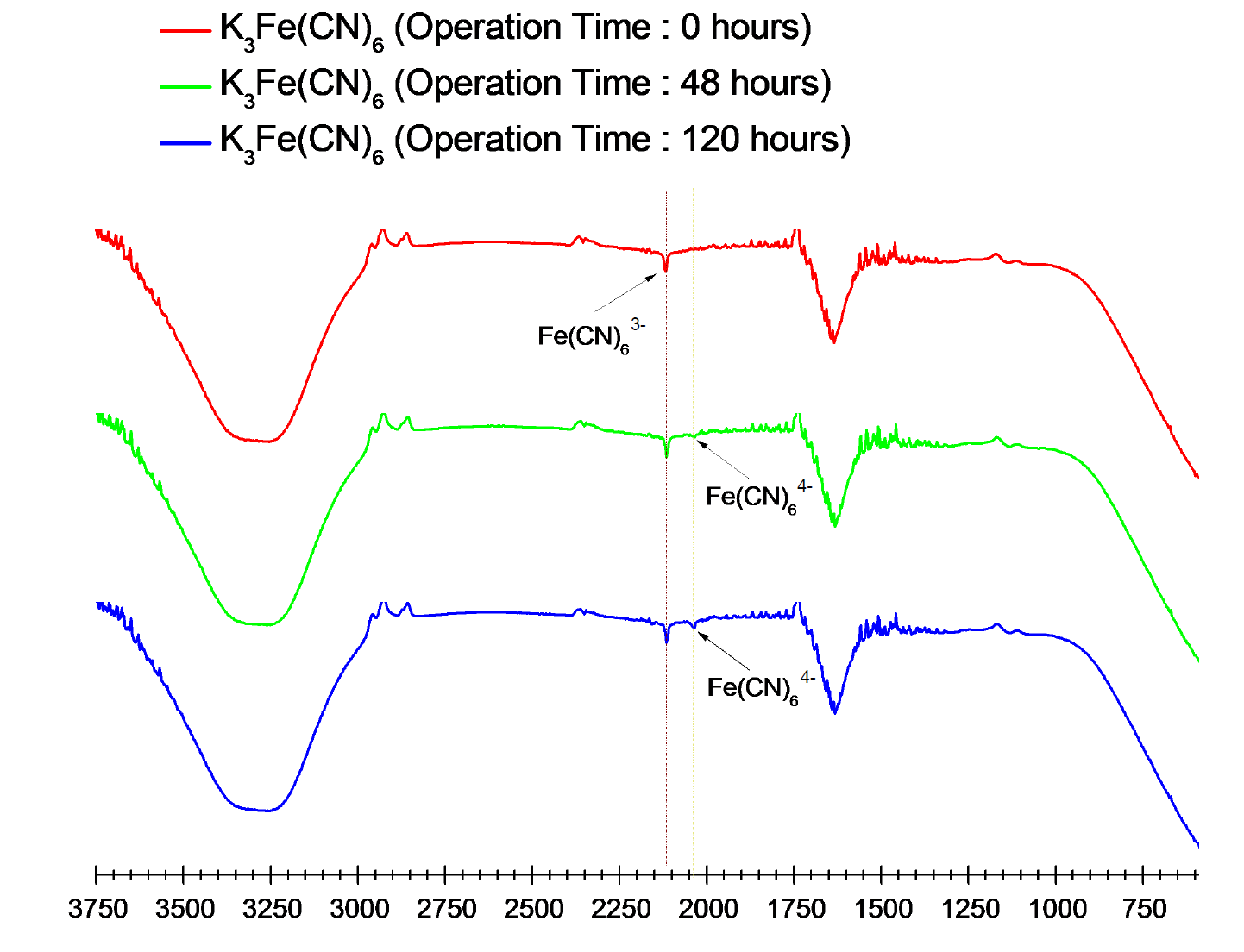


**Fig. S3.**

FTIR spectra (cm^-1^) from K_3_Fe(CN)_6_ solution at the beginning, 48-hour and 120-hour benchmark depicting slow formation of Fe(CN)_6_^4-^ over time under temperature gradient.

**Fig. S4.**

FTIR spectra (cm^-1^) from the optimized electrolyte solution on 48-hour and 120-hour time stamps.

**
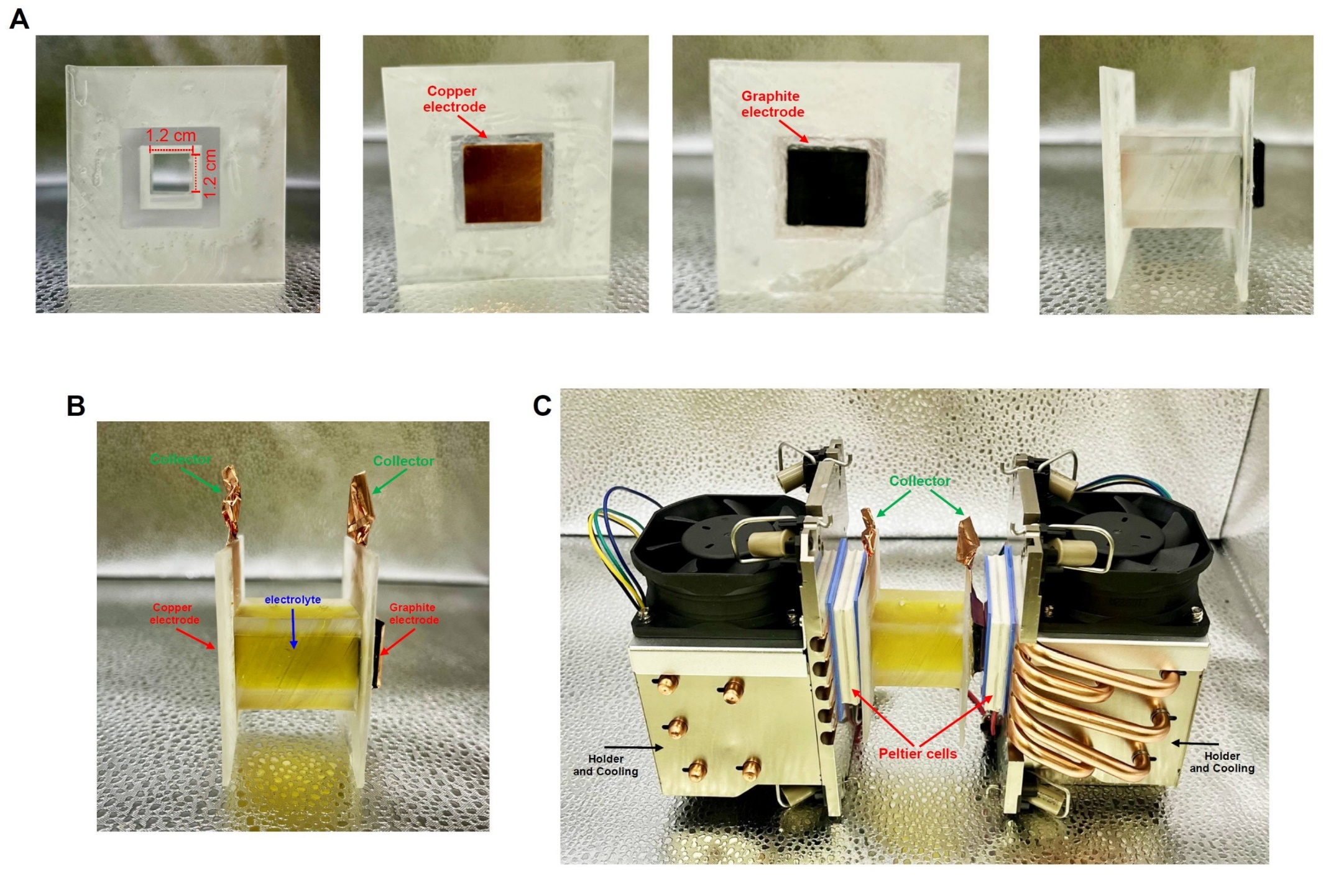
**

**Fig. S5.** (A) Printed resin cell in different views and assembled electrodes on each side. (B) Assembled cell with operational situation, electrodes connected to collectors, and electrolyte added into the cell. (C) Cell with an operational setup, connected to Peltier cells and supported by a standing fan.

**Fig. S6.**

Raman spectroscopy demonstrating the cooperative role of NH_4_OH and CH_3_COOH in sustaining the Fe(CN)_6_^3-^/^4-^ redox balance.

**Fig. S7.**

MD simulation box: (A) electrolyte model and its components (B) RDF between the Fe center of Fe(CN)_6_^3-^ and the oxygen atoms of H_2_O. (C) RDF between the Fe center of Fe(CN)_6_^4-^ and the oxygen atoms of H_2_O.

**Fig. S8.**

DFT simulation results: (A) NH_4_^+^ interacting with Fe(CN)_6_^3-/4-^ (B) NH_4_^+^ and acetate interacting with Fe(CN)_6_^3-/4-^.


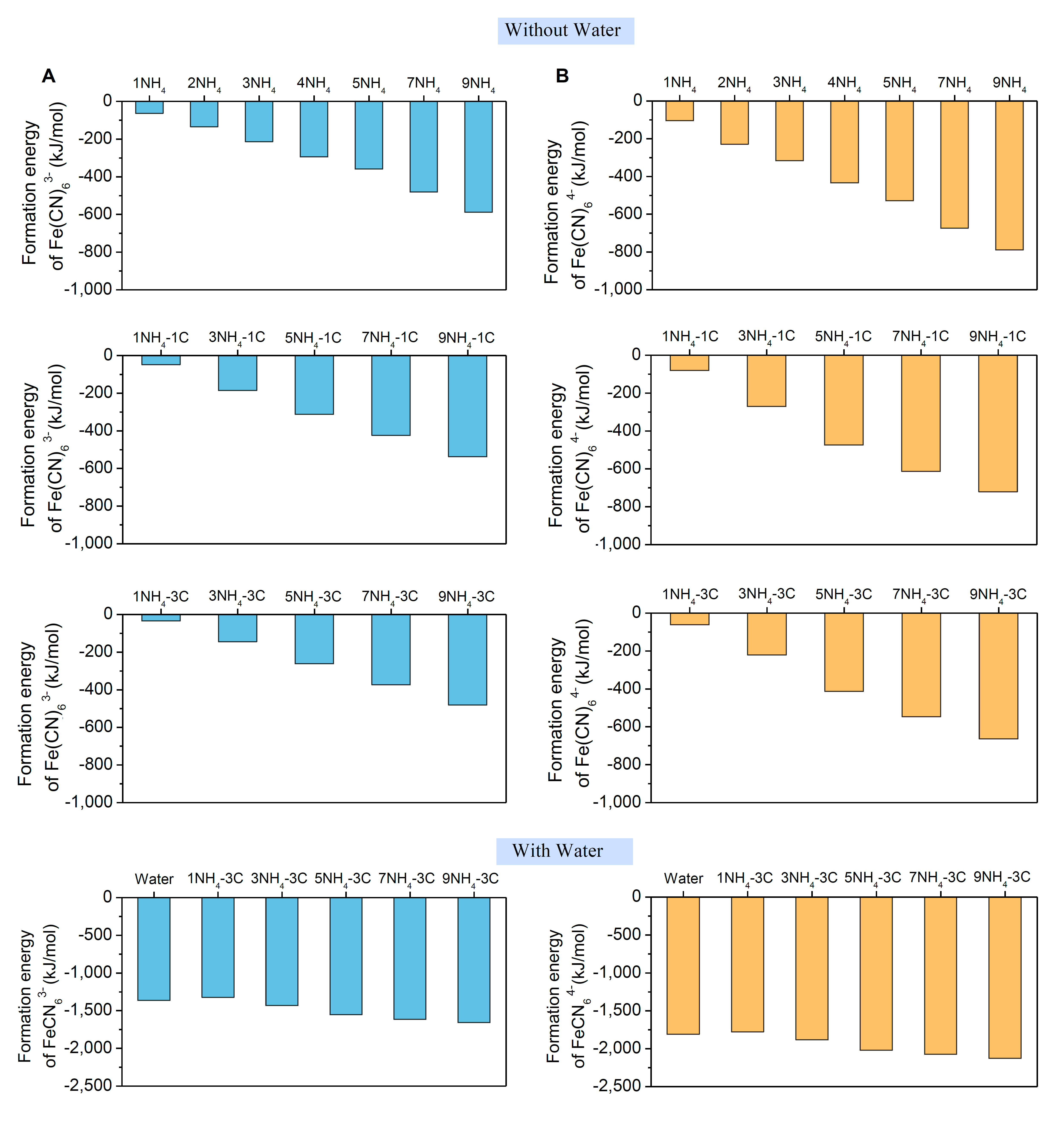


**Fig. S9.**

DFT simulation results: (A) formation energy of NH_4_^+^/acetate – Fe(CN)_6_^3-^ (B) formation energy of NH_4_^+^/acetate – Fe(CN)_6_^4-^.

**Fig. S10.**

(A) Effective electrical conductivity/resistance of several compositions of electrolyte (B) Effective electrical conductivity over a range of temperature from 298 to 303 K. (C) Effective thermal conductivity over a range of temperature from 298 to 303 K.


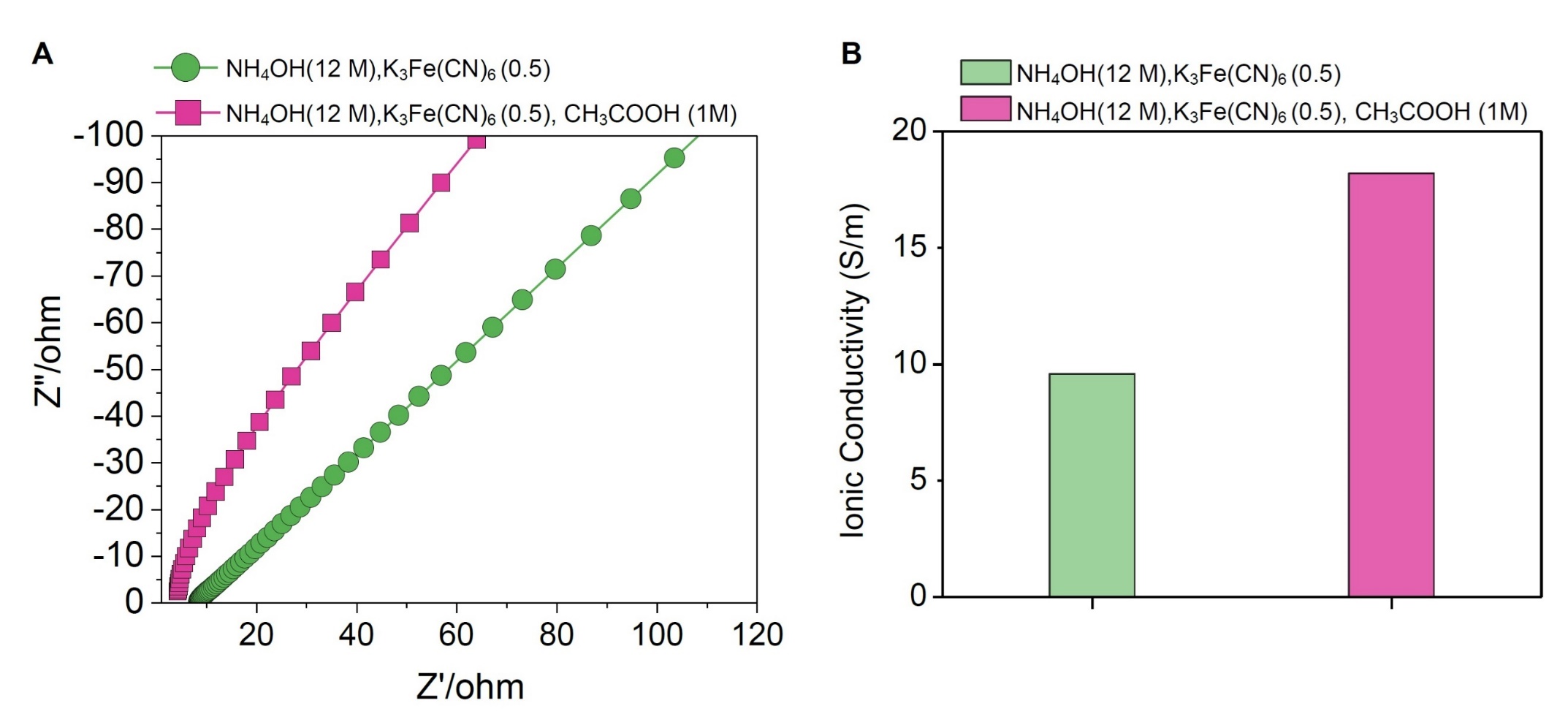


**Fig. S11.**

(A) Nyquist plot and (B) Ionic conductivity for the cell operating with electrolyte with and without CH_3_COOH.

**Fig. S12.**

Performance under a 10 Ω load at ΔT = 30 K, highlighting continuous and stable power delivery maintained over extended operation.

**Fig. S13.**

Galvanic V_oc_ measured against graphite counter electrode (No temperature difference applied).

**Fig. S14.**

(A) Open Circuit: This diagram illustrates the mechanism in an open circuit, where ions and molecules are positioned without current flow through the load. (B) Closed Circuit: This diagram shows the mechanism in a closed circuit, where a stable current is maintained through continuous redox cycling of Fe(CN)_6_^4-^ and Fe(CN)_6_^3-^.


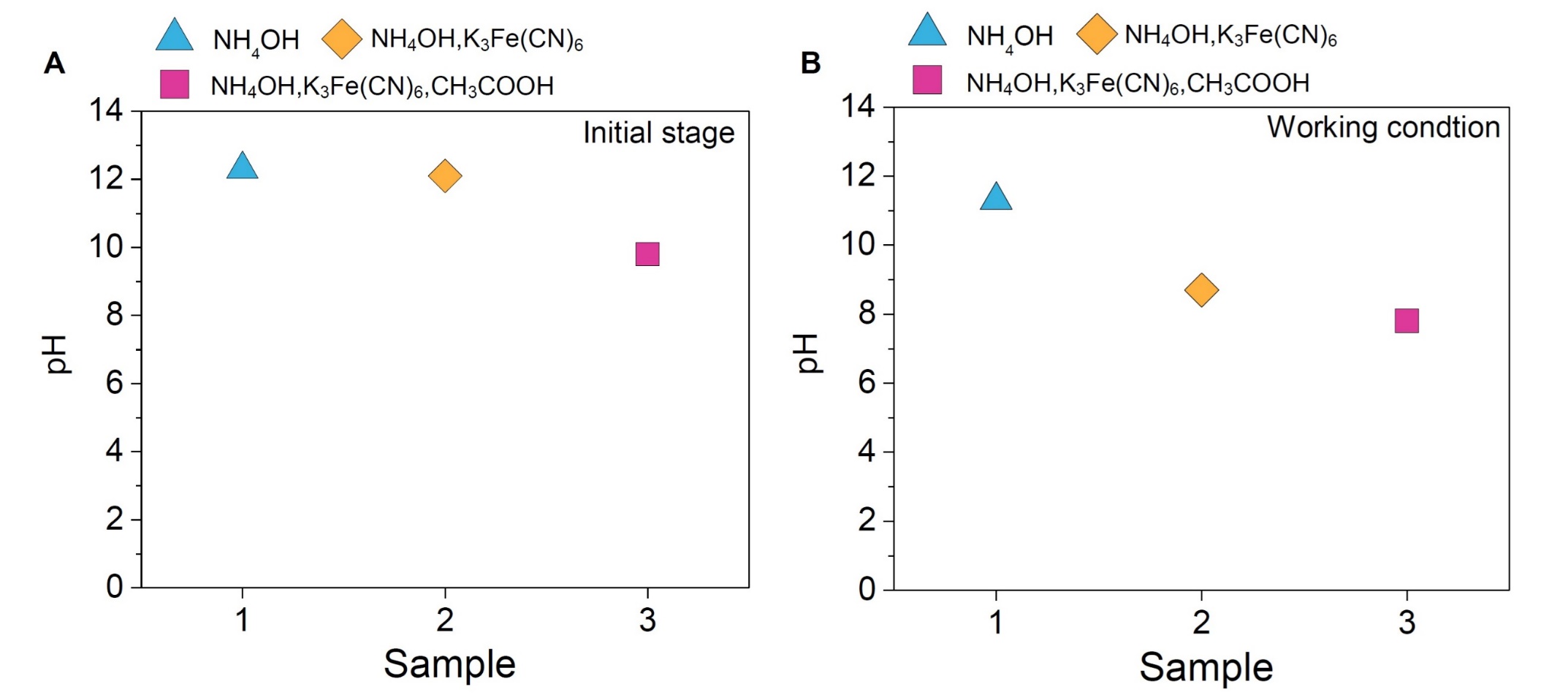


**Fig. S15.**

pH of the electrolyte mixture: NH_4_OH base, NH_4_OH-K_3_Fe(CN)_6_ mixture and final NH_4_OH-K_3_Fe(CN)_6_-CH_3_COOH electrolyte in (A) the initial stage and (B) after 30 hours of operation.


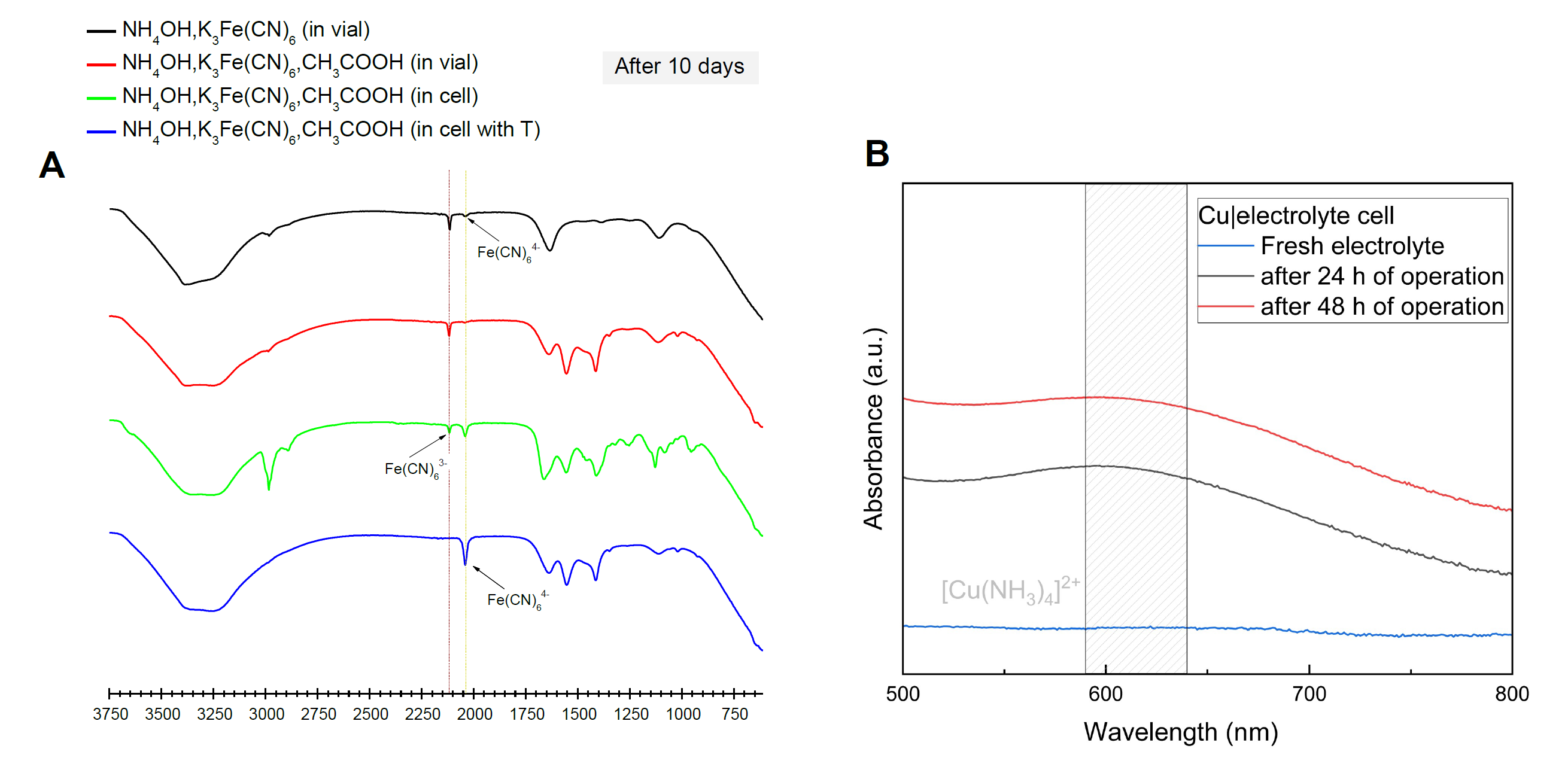


**Fig. S16.** (A) FTIR results (cm^-1^): (black) NH_4_OH/K_3_Fe(CN)_6_ and (red) NH_4_OH/K_3_Fe(CN)_6_/acetic acid. (green), NH_4_OH/K_3_Fe(CN)_6_/acetic acid and (blue) NH_4_OH/K_3_Fe(CN)_6_/acetic acid after 10 days of performance. (B) UV-vis spectrum of the fresh electrolyte, compared to the samples from the electrolyte after 24 hours and 48 hours of operation. The absorbance near 600 nm corresponds to the [Cu(NH_3_)_4_]^2+^ ammine complex, which increases with operation time.

**Fig. S17.**

Thermally-augmented galvanic contribution to the voltage per temperature gradient applied observed in the Cu| electrolyte |graphite setup with base electrolyte fabricated with different materials under variable temperature difference.

**Fig. S18.** The effect of including CH_3_COOH over maximum power density in isothermal and non-isothermal (ΔT = 10 K) conditions.

**
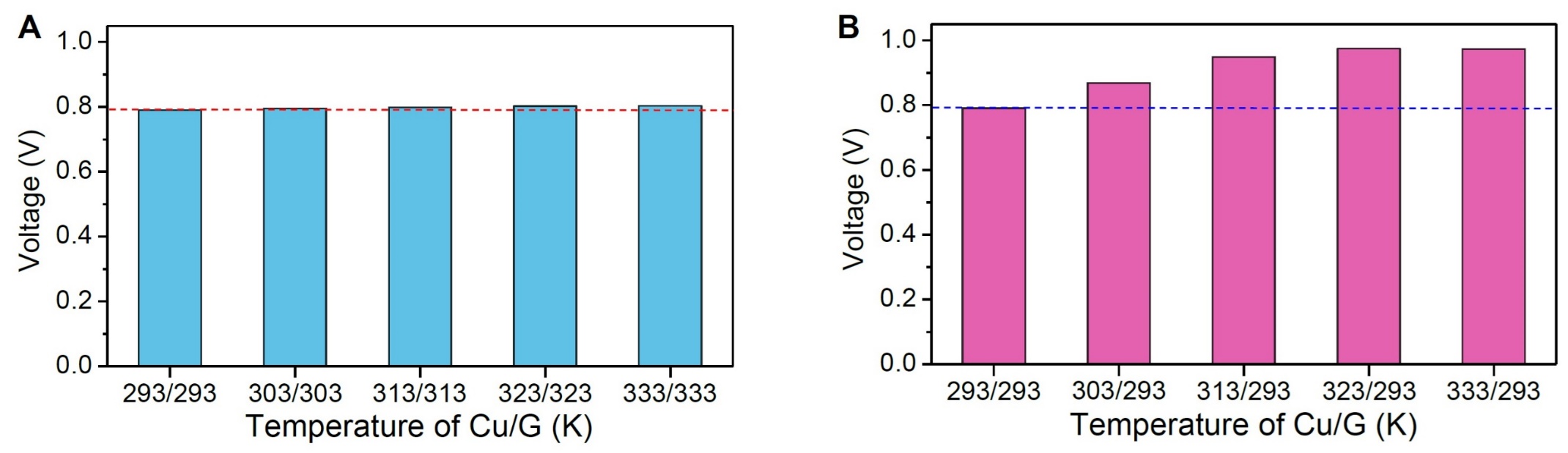
**

**Fig. S19.**

(A) Total voltage of the device in isothermal conditions depicted against electrode temperature. (B) Total voltage of the device under temperature gradient, with the cold electrode kept at 293 K, and the hot electrode temperature rising from 293 K to 333 K.

**Fig. S20.**

(A) Net voltage generated from increasing the temperature of both electrodes over 293 K. (B) Net voltage generated from increasing the copper electrode temperature over 293 K.

**Fig. S21.**

Symmetric-cell control measurements isolating the contributions to the asymmetric gTg total thermal response. Voltage and Thermopower time series of Cu|electrolyte|Cu cell configurations.

**Fig. S22.**

Voltage and current output corresponding to different electrolytes during the charging and discharging under 333 kΩ.


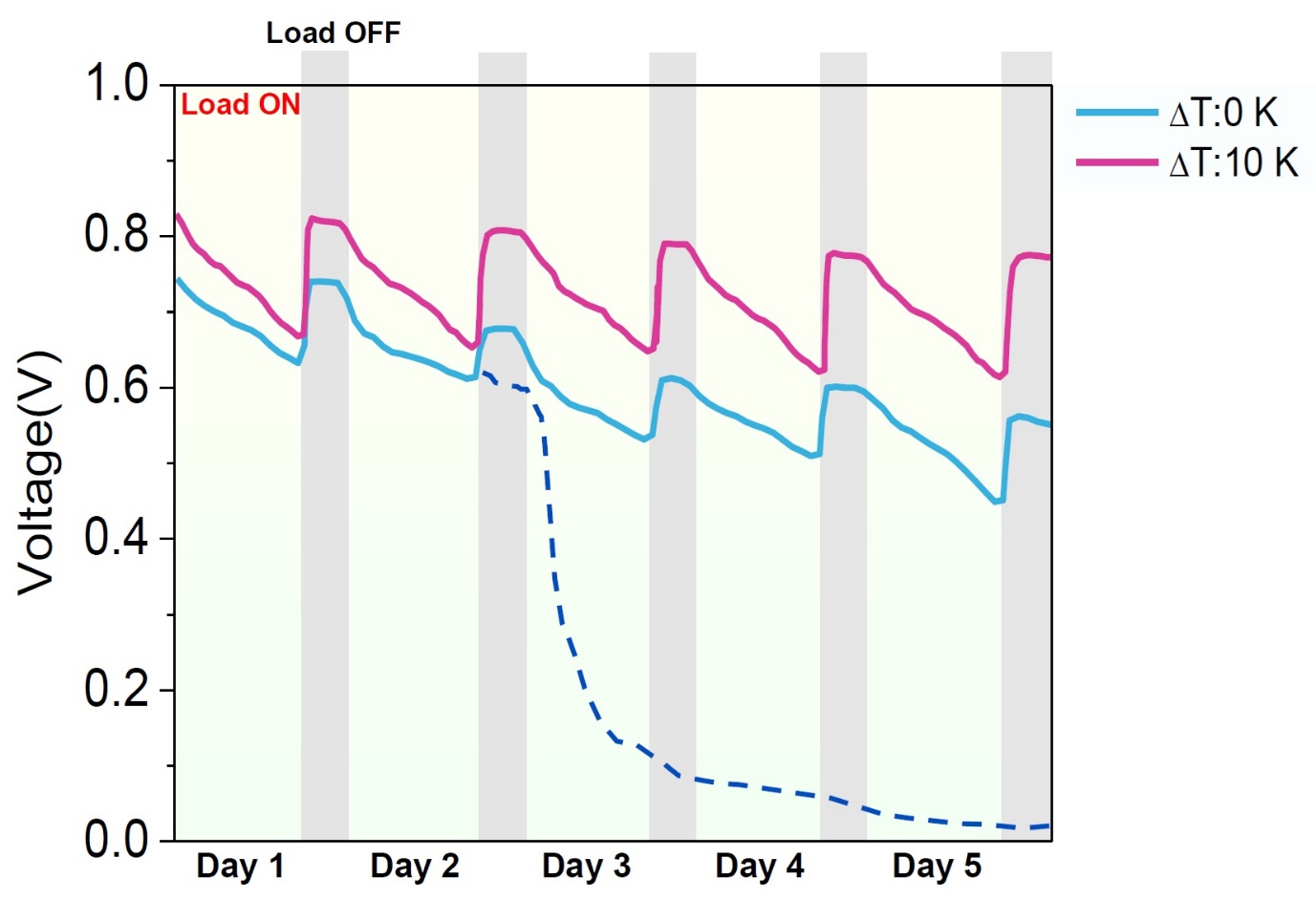


**Fig. S23.**

Voltage measurements over long discharge period (five days) observed for the cell working under the isothermal and the applied temperature gradient (ΔT = 10 K). The dashed line shows the operating voltage of the device working under isothermal conditions if the container is not gently shaken before initiating each cycle.


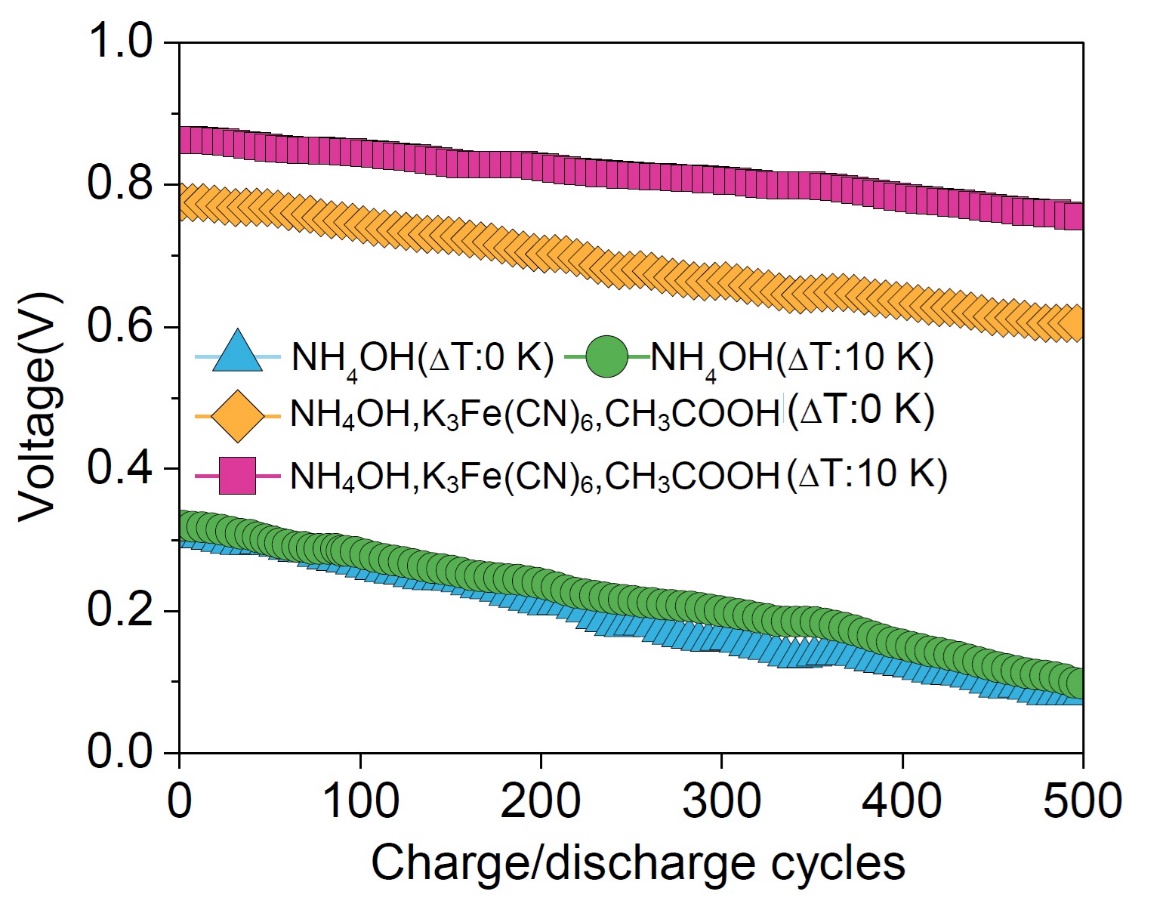


**Fig. S24.** Working voltage of first 500 cycles for the prototype Copper | electrolyte | graphite cell with different electrolyte mixtures.


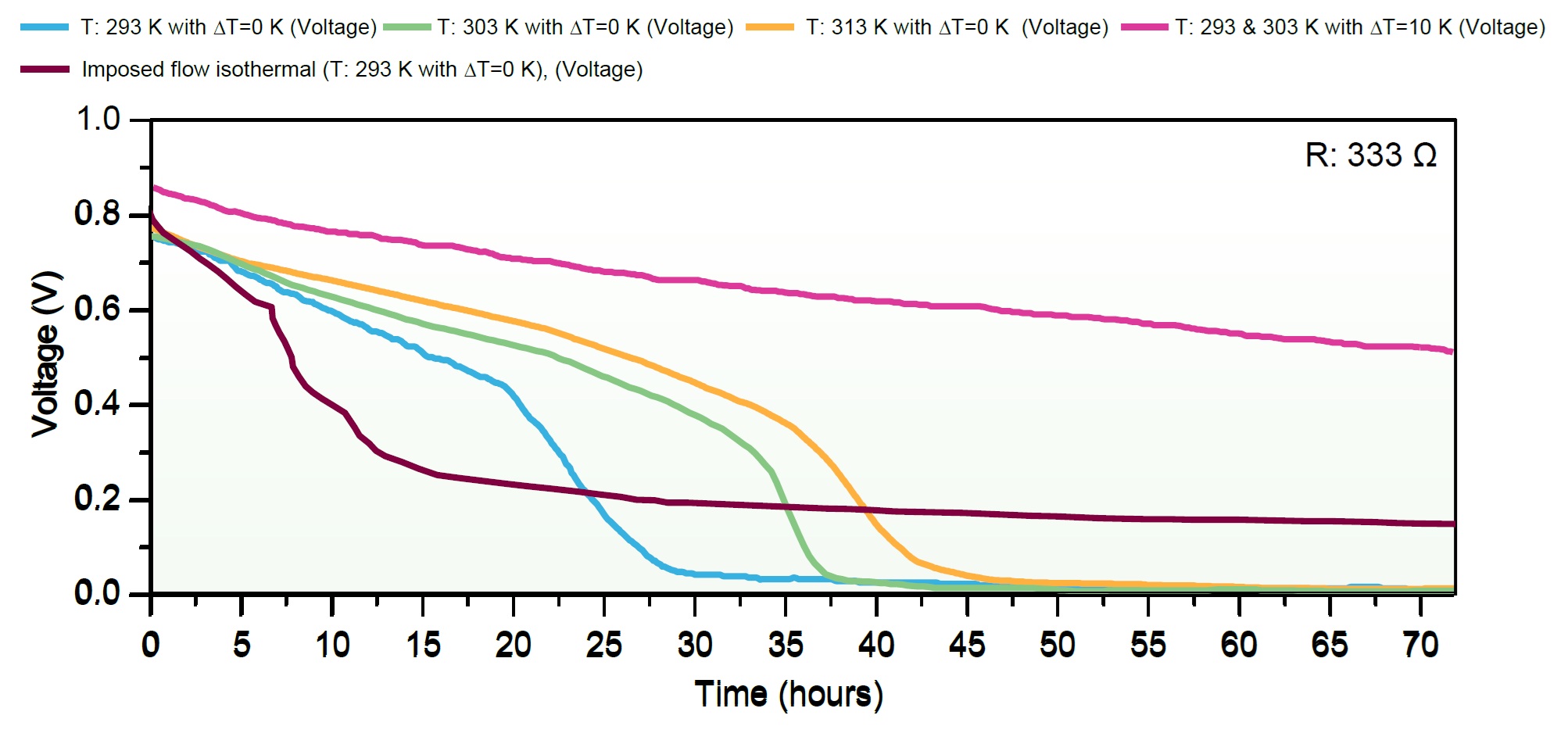


**Fig. S25.**

Closed-circuit voltage for isothermal (293 K, 303 K and 313 K) and gTg (ΔT = 10 K) cells tracked over 70 hours. We also tracked the effect of imposed flow in isothermal conditions (293 K).

Table S2. Comparison of thermopower (S_e_),normalized power density (P_max_/ΔT^2^) and Carnot-relative efficiency (η_r_) from this study with values reported in the literature for thermogalvanic cells.

*Values marked with an asterisk represent the total thermal voltage enhancement coefficient and normalized power density of the asymmetric Cu|electrolyte|G (gTg) architecture, which includes both thermogalvanic and thermally-augmented galvanic contributions. These are not directly comparable to the intrinsic thermopower of the symmetric thermogalvanic cells listed in this table.

| Matrix | Redox couple | $S_{e}$  (mV K^-1^) | $P_{max}/\left( \Delta T \right)^{2}$ (mW m^-2^ K^-2^) | $\eta_{r}$ (%) | Ref. |
| --- | --- | --- | --- | --- | --- |
| NH_4_OH-CH_3_COOH (gTg) | FeCN^4-^/^3-^ | 7.81* | 40.6* | 50.55 | This work |
| NH_4_OH-CH_3_COOH (Tg) | FeCN^4-^/^3-^ | 2.64 | 1.5 | 2.5 | This work |
| Ov–WO3/PAA/Sv–ZIS | FeCN^4-^/^3-^ | 8.2 | 8.5 | 4.91 | [19] |
| H_2_O | FeCN^4-^/^3-^ | 3.7 | - | 14.8 | [20] |
| H_2_O | FeCN^4-^/^3-^ | 3.73 | 7.08 | 11.1 | [21] |
| PVA | FeCN^4-^/^3-^ | 5.81 | 11.90 | 8.53 | [22] |
| PAA-sodium alginate | FeCN^4-^/^3-^ | 4.4 | 1.78 | 3.05 | [23] |
| polyacrylamide | FeCN^4-^/^3-^ | 1.5 | 0.61 | 1.38 | [24] |
| H_2_O | FeCN^4-^/^3-^ | 2.9 | 0.64 | 1.38 | [25] |
| H_2_O | FeCN^4-^/^3-^ | 1.42 | 1.9 | 2.4 | [26] |
| polyacrylamide | Fe^3+^/^2+^ | 2.91 | 0.66 | - | [27] |
| H_2_O | FeCN^4-^/^3-^ | 4.2 | 1.91 | - | [28] |
| polyvinyl alcohol | Fe^3+^/^2+^ | 2.02 | 0.1 | - | [29] |
| H_2_O | FeCN^4-^/^3-^ | 1.43 | 0.2 | 0.275 | [30] |
| H_2_O | FeCN^4-^/^3-^ | 1.3 | 0.36 | 0.7 | [31] |
| PAA | FeCN^4-^/^3-^ | 2.7 | 0.82 | 0.47 | [19] |
| tetramethylene sulfone | Fe^3+^/^2+^ | 2.49 | 0.02 | - | [32] |
| polyvinyl alcohol/gelation | Fe^3+^/^2+^ | 1.63 | 0.03 | - | [33] |
| PAA-carboxymethylcellulose | FeCN^4-^/^3-^ | 1.3 | 0.0325 | - | [34] |
| polyvinyl alcohol | Fe^3+^/^2+^ | 0.79 | 0.05 | - | [35] |
| α-cyclodextrin | I^-^/I^3-^ | 2.0 | 0.052 | - | [36] |
| H_2_O | Co^3+^/^2+^ | 1.6 | 0.06 | - | [37] |
| H_2_O | FeCN^4-^/^3-^ | 1.45 | 0.12 | - | [38] |
| polyvinyl alcohol | FeCN^4-^/^3-^ | 1.5 | 0.17 | - | [39] |
| H_2_O | Fe^3+^/^2+^ | 1.70 | 0.48 | - | [40] |
| H_2_O | FeCN^4-^/^3-^ | 1.4 | 0.5 | - | [41] |
| H_2_O | Fe^3+^/^2+^ | 1.72 | 0.56 | - | [42] |
| H_2_O | FeCN^4-^/^3-^ | 1.43 | 0.6 | 0.4 | [43] |
| H_2_O | Cu^2+^/Cu^+^ | 1.66 | 0.71 | - | [44] |

**References**

1. Prampolini, G., et al., *Structure and dynamics of ferrocyanide and ferricyanide anions in water and heavy water: An insight by MD simulations and 2D IR spectroscopy.* The Journal of Physical Chemistry B, 2014. **118**(51): p. 14899-14912.

2. Rappe, A., K. Colwell, and C. Casewit, *Application of a universal force field to metal complexes.* Inorganic Chemistry, 1993. **32**(16): p. 3438-3450.

3. Rappé, A.K., et al., *UFF, a full periodic table force field for molecular mechanics and molecular dynamics simulations.* Journal of the American chemical society, 1992. **114**(25): p. 10024-10035.

4. Martyna, G.J., M.L. Klein, and M. Tuckerman, *Nosé–Hoover chains: The canonical ensemble via continuous dynamics.* The Journal of chemical physics, 1992. **97**(4): p. 2635-2643.

5. Kresse, G. and J. Furthmüller, *Efficiency of ab-initio total energy calculations for metals and semiconductors using a plane-wave basis set.* Computational materials science, 1996. **6**(1): p. 15-50.

6. Blöchl, P.E., *Projector augmented-wave method.* Physical review B, 1994. **50**(24): p. 17953.

7. Grimme, S., et al., *A consistent and accurate ab initio parametrization of density functional dispersion correction (DFT-D) for the 94 elements H-Pu.* The Journal of chemical physics, 2010. **132**(15).

8. Grimme, S., S. Ehrlich, and L. Goerigk, *Effect of the damping function in dispersion corrected density functional theory.* Journal of computational chemistry, 2011. **32**(7): p. 1456-1465.

9. Le Caër, S., et al., *First coupling between a LINAC and FT-IR spectroscopy: The aqueous ferrocyanide system.* Chemical physics letters, 2006. **426**(1-3): p. 71-76.

10. Zhang, J., et al., *In situ fourier transform infrared reflection spectroscopic studies of ferricyanide/ferrocyanide on graphite electrode.* Electroanalysis, 1993. **5**(5‐6): p. 517-520.

11. Hu, M., et al., *Cycling performance and mechanistic insights of ferricyanide electrolytes in alkaline redox flow batteries.* Advanced Energy Materials, 2023. **13**(15): p. 2203762.

12. Xia, M., et al., *Hydrogen bond chemistry in Fe4 [Fe (CN) 6] 3 host for aqueous NH4+ batteries.* Chemical Engineering Journal, 2021. **421**: p. 127759.

13. Liu, W., et al., *Construction and application of thermogalvanic hydrogels.* Soft Science, 2024. **4**(4): p. N/A-N/A.

14. Vanysek, P., *Ionic conductivity and diffusion at infinite dilution.* CRC hand book of chemistry and physics, 1993: p. 5-92.

15. Zhang, F., et al., *A thermally regenerative ammonia-based battery for efficient harvesting of low-grade thermal energy as electrical power.* Energy & Environmental Science, 2015. **8**(1): p. 343-349.

16. Reber, D., et al., *Stability of highly soluble ferrocyanides at neutral pH for energy-dense flow batteries.* Cell Reports Physical Science, 2023. **4**(1).

17. Pourbaix, M., *Atlas of electrochemical equilibria in aqueous solutions.* (No Title), 1974.

18. Beverskog, B. and I. Puigdomenech, *Revised Pourbaix diagrams for copper at 25 to 300 C.* Journal of the Electrochemical Society, 1997. **144**(10): p. 3476-3483.

19. Wang, Y., et al., *In situ photocatalytically enhanced thermogalvanic cells for electricity and hydrogen production.* Science, 2023. **381**(6655): p. 291-296.

20. Zhuang, X., et al., *Self‐Assembled Asymmetric Electrodes for High‐Efficiency Thermogalvanic Cells.* Advanced Energy Materials, 2023. **13**(39): p. 2302011.

21. Yu, B., et al., *Thermosensitive crystallization–boosted liquid thermocells for low-grade heat harvesting.* Science, 2020. **370**(6514): p. 342-346.

22. Wang, J., et al., *Ultrastrong, flexible thermogalvanic armor with a Carnot-relative efficiency over 8%.* Nature Communications, 2024. **15**(1): p. 6704.

23. Zhang, D., et al., *Stretchable thermogalvanic hydrogel thermocell with record-high specific output power density enabled by ion-induced crystallization.* Energy & Environmental Science, 2022. **15**(7): p. 2974-2982.

24. Lei, Z., W. Gao, and P. Wu, *Double-network thermocells with extraordinary toughness and boosted power density for continuous heat harvesting.* Joule, 2021. **5**(8): p. 2211-2222.

25. Kim, T., et al., *High thermopower of ferri/ferrocyanide redox couple in organic-water solutions.* Nano Energy, 2017. **31**: p. 160-167.

26. Romano, M.S., et al., *Carbon nanotube-reduced graphene oxide composites for thermal energy harvesting applications.* 2013.

27. Xu, C., et al., *Adaptable and wearable thermocell based on stretchable hydrogel for body heat harvesting.* Advanced Energy Materials, 2022. **12**(42): p. 2201542.

28. Duan, J., et al., *Aqueous thermogalvanic cells with a high Seebeck coefficient for low-grade heat harvest.* Nature communications, 2018. **9**(1): p. 5146.

29. Gao, W., et al., *Stretchable and freeze‐tolerant organohydrogel thermocells with enhanced thermoelectric performance continually working at subzero temperatures.* Advanced Functional Materials, 2021. **31**(43): p. 2104071.

30. Kang, T.J., et al., *Electrical power from nanotube and graphene electrochemical thermal energy harvesters.* Advanced Functional Materials, 2012. **22**(3): p. 477-489.

31. Li, G., et al., *High‐efficiency cryo‐thermocells assembled with anisotropic holey graphene aerogel electrodes and a eutectic redox electrolyte.* Advanced Materials, 2019. **31**(25): p. 1901403.

32. Liu, Y., et al., *Solvent effect on the Seebeck coefficient of Fe 2+/Fe 3+ hydrogel thermogalvanic cells.* Journal of Materials Chemistry A, 2022. **10**(37): p. 19690-19698.

33. Bai, C., et al., *Transparent stretchable thermogalvanic PVA/gelation hydrogel electrolyte for harnessing solar energy enabled by a binary solvent strategy.* Nano Energy, 2022. **100**: p. 107449.

34. Shen, J., et al., *Boosting solar-thermal-electric conversion of thermoelectrochemical cells by construction of a carboxymethylcellulose-interpenetrated polyacrylamide network.* Journal of Materials Chemistry A, 2022. **10**(14): p. 7785-7791.

35. Bai, C., et al., *Wearable electronics based on the gel thermogalvanic electrolyte for self-powered human health monitoring.* ACS Applied Materials & Interfaces, 2021. **13**(31): p. 37316-37322.

36. Hongyao, Z., Y. Teppei, and K. Nobuo, *Supramolecular Thermo-Electrochemical Cells: Enhanced Thermoelectric Performance by Host–Guest Complexation and Salt-Induced Crystallization.* 2016.

37. Salazar, P.F., et al., *Enhanced thermo-electrochemical power using carbon nanotube additives in ionic liquid redox electrolytes.* Journal of materials chemistry a, 2014. **2**(48): p. 20676-20682.

38. Yang, Y., et al., *Charging-free electrochemical system for harvesting low-grade thermal energy.* Proceedings of the National Academy of Sciences, 2014. **111**(48): p. 17011-17016.

39. Gao, W., et al., *Hierarchically anisotropic networks to decouple mechanical and ionic properties for high-performance quasi-solid thermocells.* ACS nano, 2022. **16**(5): p. 8347-8357.

40. Kim, J.H. and T.J. Kang, *Diffusion and current generation in porous electrodes for thermo-electrochemical cells.* ACS applied materials & interfaces, 2019. **11**(32): p. 28894-28899.

41. Hu, R., et al., *Harvesting waste thermal energy using a carbon-nanotube-based thermo-electrochemical cell.* Nano letters, 2010. **10**(3): p. 838-846.

42. Lee, J.H., et al., *An electricity-generating window made of a transparent energy harvester of thermocells.* ACS applied materials & interfaces, 2021. **13**(18): p. 21157-21165.

43. Zhang, L., et al., *High power density electrochemical thermocells for inexpensively harvesting low-grade thermal energy.* 2017.

44. Yu, B., et al., *Cost-effective n-type thermocells enabled by thermosensitive crystallizations and 3D multi-structured electrodes.* Nano Energy, 2022. **93**: p. 106795.
